# Consistency Theory of General Nonparametric Classification Methods in Cognitive Diagnosis

**DOI:** 10.1017/psy.2025.9

**Published:** 2025-03-17

**Authors:** Chengyu Cui, Yanlong Liu, Gongjun Xu

**Affiliations:** 1 Department of Statistics, University of Michigan, Ann Arbor, MI, USA; 2 Booth School of Business, University of Chicago, Chicago, IL, USA

**Keywords:** cognitive diagnosis, consistency theory, general nonparametric classification method, Q-matrix

## Abstract

Cognitive diagnosis models (CDMs) have been popularly used in fields such as education, psychology, and social sciences. While parametric likelihood estimation is a prevailing method for fitting CDMs, nonparametric methodologies are attracting increasing attention due to their ease of implementation and robustness, particularly when sample sizes are relatively small. However, existing consistency results of the nonparametric estimation methods often rely on certain restrictive conditions, which may not be easily satisfied in practice. In this article, the consistency theory for the general nonparametric classification method is reestablished under weaker and more practical conditions.

## 1 Introduction

Cognitive diagnosis models (CDMs), also known as diagnostic classification models (DCMs), are a popular family of discrete latent variable models employed in diagnostic assessments to provide detailed information about subjects’ latent attributes based on their responses to designed diagnostic items. For instance, in educational testing, these latent attributes might indicate if a subject has mastered certain skills or not (de la Torre, 2011; Henson et al., 2009; Junker & Sijtsma, 2001); in psychiatric diagnosis, the latent attributes might signal the presence or absence of certain mental disorders (de la Torre et al., 2018; Templin & Henson, 2006).

Parametric models for cognitive diagnosis have been developed and widely applied in practice. Popular examples include the deterministic input, noisy “and” gate (DINA) model (Junker & Sijtsma, 2001), the deterministic input, noisy “or” gate (DINO) model (Templin & Henson, 2006), the general diagnostic model (GDM; von Davier, 2008), the reduced reparameterized unified model (reduced RUM; Hartz, 2002), the log-linear CDM (LCDM; Henson et al., 2009), and the generalized DINA model (GDINA; de la Torre, 2011). In conventional settings with a fixed number of items (*J*) and a large number of subjects (*N*), the latent attributes are often viewed as random variables. The corresponding CDMs can thus be viewed as a family of finite mixture models, where each subject’s latent attribute profile 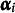 behaves as a discrete random variable following a categorical distribution. From this perspective, the estimation often takes place through the maximization of the marginal likelihood, relying on methods such as the expectation-maximization algorithm (de la Torre, 2011; DiBello et al., 2007; von Davier, 2008). However, the maximum likelihood-based approach often necessitates sufficiently large assessments to guarantee the reliability of the item parameter estimation, and it may either produce inaccurate estimates with small sample sizes or suffer from high computational costs (Chiu & Köhn, 2019a; Chiu et al., 2018). Moreover, the parametric CDMs involve certain parametric assumptions about the item response functions, which may raise concerns about the validity of the assumed model and the underlying process (Chiu & Douglas, 2013).

As an alternative, researchers have explored nonparametric cognitive diagnosis methods (Chiu & Köhn, 2019b; Chiu et al., 2009). Instead of modeling the item response functions parametrically, the nonparametric methods aim to directly categorize subjects into latent groups by minimizing certain distance measure between a subject’s observed item responses and some expected “centers” of the latent groups. Two popular examples of nonparametric cognitive diagnosis methods include the nonparametric classification (NPC) method (Chiu & Douglas, 2013) and its generalization, the general NPC (GNPC) method (Chiu et al., 2018). The GNPC method, in particular, has received increasing attention in recent years due to its effectiveness in handling complex CDMs and its good performance for sample sizes (Chandía et al., 2023; Chiu & Chang, 2021; Ma, de la Torre, et al., 2023; Wang et al., 2023). The algorithms of the NPC and GNPC methods are straightforward to implement and require minimal computational resources, making them highly appealing for practical applications.

Theoretical properties of the nonparametric methods have also been explored in the literature. Under some regularity conditions, the NPC estimators of the subjects’ latent attribute profiles have been shown to be statistically consistent for certain CDMs, including DINA and reduced RUM (Wang & Douglas, 2015), and a similar consistency theory for the GNPC estimator has also been established (Chiu & Köhn, 2019a). However, the current theoretical guarantees for these nonparametric methods depend on relatively stringent assumptions. In the case of the NPC method, the assumptions associated with the ideal binary responses might oversimplify the underlying diagnostic process and thus be challenging to fulfill when dealing with complex underlying CDMs, such as the GDINA model and other general CDMs (Chiu et al., 2018). Although the GNPC method addresses the oversimplification issue of the NPC method, its consistency depends on a key assumption that consistent initial estimators of the latent attribute profiles are available. For instance, Theorem 1 in Chiu and Köhn (2019a) provides theoretical guarantees for the GNPC estimators, assuming an initialization that consistently estimates the ground truth latent memberships. Similarly, Theorems 1–3 in Ma, de la Torre, et al. (2023) require consistent estimation of latent memberships from a calibration dataset to establish their consistency results. The assumption that consistent initial estimators of latent attribute profiles can be obtained or that a calibration dataset is available may be overly restrictive in practice, and the consistency of the GNPC method in more realistic settings remains an open problem.

In this article, we establish the consistency for the GNPC method using different theoretical techniques, without relying on the previous assumption on initial consistent estimators or calibration datasets. Our analysis covers both the original GNPC method in Chiu and Köhn (2019a) and a modified version of the GNPC method in Ma, de la Torre, et al. (2023).

We establish finite-sample error bounds for latent attributes of general nonparametric methods as well as uniform consistency of the item parameters. We would like to clarify that the main contribution of this work lies in the theoretical analysis of the GNPC and modified GNPC methods. For the implementation of these methods, we recommend utilizing the algorithms proposed in the literature (Chiu & Köhn, 2019a; Chiu et al., 2018; Ma, de la Torre, et al., 2023), which have demonstrated the effectiveness of GNPC methods via extensive simulation studies and real data examples.

The rest of the paper is organized as follows: Section 2 provides a brief review of cognitive diagnostic models and discusses the limitations in the existing consistency results. Section 3 establishes consistency results of the GNPC methods. In Section 4, we provide a simulation study to illustrate our theoretical results. Section 5 gives some further discussions, and the Supplementary Material provides the proofs for the main results.

## 2 Model setup and nonparametric methods

This work focuses on CDMs for multivariate binary data, which are commonly encountered in educational assessments (correct/wrong answers) and social science survey responses (yes/no responses) (von Davier & Lee, 2019). For *N* subjects and *J* items, the observed data is an 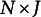 binary matrix 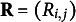 , where 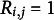 or 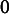 denotes whether the *i*th subject gives a positive response to the *j*th item. Consider *K* binary latent attributes. Let the row vector 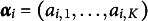 represent the latent attribute profile for the *i*th subject, where 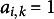 or 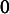 indicates the presence or absence, respectively, of the *k*th attribute for the *i*th individual. We further use an 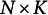 binary matrix, 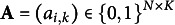 , to represent the latent attribute profiles for all *N* subjects.

To capture the dependence relationship between items and the latent attributes of subjects, a design matrix called the Q-matrix (Tatsuoka, 1985) is employed. The Q-matrix encodes how the *J* items depend on the *K* latent attributes. Specifically, 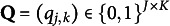 , where 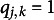 or 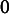 indicates whether the *j*th test item depends on the *k*th latent attribute, and we denote the *j*th item’s Q-matrix vector as 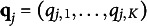 .

For an integer *m*, we denote 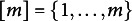 and for a set 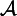 , we denote its cardinality by 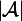 . We denote 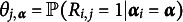 for any 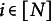 , 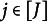 and 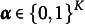 , and let 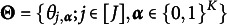 . We assume each response 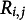 follows a Bernoulli distribution with parameter 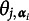 and the responses are independent with each other conditional on the latent attribute profiles 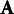 and the structure loading matrix 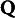 . In summary, the data generative process aligns with the following latent class model: 

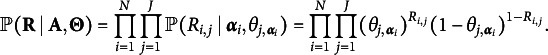

To further illustrate the adaptability of the general nonparametric method to the model structures embedded in CDMs imposed by the structural matrix 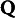 , we follow the general assumption for the restricted latent class models (Chiu & Köhn, 2015; Ma, de la Torre, et al., 2023; Xu, 2017) that for different attribute profiles 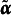 and 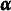 , (1)

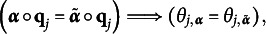

where 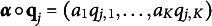 denotes the element-wise product of binary vectors 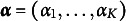 and 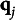 . This implies that the item response parameter 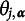 only depends on whether the latent attribute profile 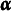 contains the required attributes 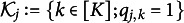 for item *j*. In cognitive diagnostic assessments, the matrix 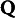 is typically predetermined by domain experts (George & Robitzsch, 2015; Junker & Sijtsma, 2001; von Davier, 2008). In this work, we assume the Q-matrix 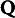 is specified, and 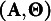 are to be estimated from the responses 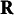 .

### 2.1 Parametric CDMs: DINA and DINO models

For parametric CDMs, the structural matrix 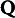 imposes various constraints on the item parameters based on different cognitive assumptions. For instance, in the DINA (Junker & Sijtsma, 2001) model, a conjunctive relationship among the attributes is assumed. According to this assumption, for a subject to provide a positive (correct) response to an item, mastery of all the required attributes of the item is necessary. In the DINA model, the ideal response for each item 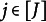 and each latent attribute profile 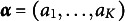 is defined as 

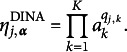



The DINO (Templin & Henson, 2006) model assumes a disjunctive relationship among attributes, where mastery of at least one of the required attributes for an item is necessary for a subject to be considered capable of providing a positive response. In the DINO model, the ideal response is defined as 

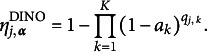



The DINA and DINO models further encompass uncertainty by incorporating the slipping and guessing parameters, denoted as 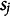 and 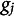 for 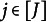 . For each item *j*, the slipping parameter represents the probability of a capable subject giving a negative response, whereas the guessing parameter signifies the probability of an incapable subject giving a positive response. Specifically, 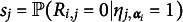 and 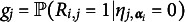 for the *i*th subject. Therefore, in these two restricted latent class models, the parameter 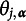 can be expressed as 

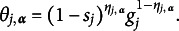



### 2.2 Nonparametric CDMs: NPC and GNPC

For nonparametric CDMs, the ideal responses described under the DINA and DINO models serve as foundational elements for the NPC analysis. Given a set of 0–1 binary ideal responses, denoted as 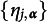 , the NPC method, as introduced by Chiu and Douglas (2013), estimates the subjects’ latent attribute profiles as follows. This method utilizes a distance-based algorithm, leveraging observed item responses to categorize subjects into latent groups. The NPC estimator, 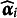 , for the *i*th individual’s attribute profile, 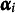 , is expressed as 

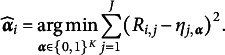



In the NPC method, the ideal responses 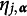 can be based on either the DINA model or the DINO model. However, due to the dependence on these specific model assumptions, which define two extreme relations between 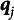 and latent attribute profile 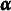 , the NPC method may fail to handle complex CDMs, such as the GDINA model, and such limitation may lead to misclassifications of the subjects (Chiu & Köhn, 2019a).

To address this issue, the GNPC method (Chiu et al., 2018) offers a solution by considering a more general ideal response that represents a weighted average of the ideal responses from the DINA and DINO models, as in (2)

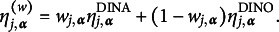



The weights are determined by the data; therefore, the proportional influence of 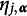 and 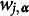 on the weighted ideal item response is adapted to the complexity of the underlying CDM data generating process. The GNPC method can be utilized with any CDM that can be represented as a general CDM, without requiring prior knowledge of the underlying model. To obtain estimates of the weights, Chiu et al. (2018) proposed minimizing the 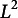 distance between the responses to item *j* and the weighted ideal responses 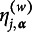 : 

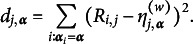

When 
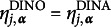
 , this results in 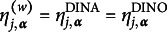 , which happens either when 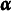 includes all the required latent attributes in 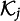 , leading to 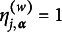 , or when 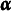 does not contain any required attributes, resulting in 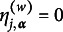 . Equivalently, these two extreme situations can be summarized as the following constraints: (3)

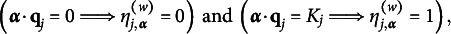

where 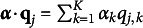 denotes the inner product of the two vectors and 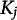 is defined as 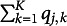 , representing the number of latent attributes that the *j*th item depends on. Thus, in these two extreme situations, the parameters 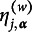 are known and do not need estimation. In scenarios where 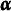 includes only some of the required attributes, 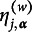 need to be estimated, and in such cases, minimizing 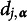 would lead to (4)

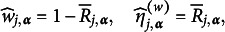

where 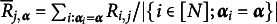 , which represents the sample mean of the responses to the *j*th item for subjects with given latent attribute profile 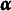 . Since the true latent attribute profiles are unknown, the memberships and the ideal responses will be jointly estimated. Specifically, the optimization problem associated with the GNPC method in Chiu et al. (2018) aims to minimize the following loss function over the membership 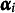 and the weights 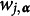 under the constraints imposed by the given Q-matrix: (5)

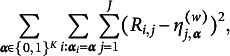

under constraint (1), where 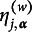 is given in (2).

A modified GNPC method was studied by Ma, de la Torre, et al. (2023) under a general framework where the item parameters 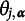 are treated as a certain “centroid.” In their framework, the item parameters 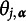 and latent attributes 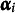 are obtained by minimizing 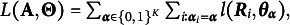 where 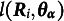 is a loss function that measures the distance between the *i*th subject’s response vector, 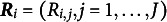 , and the item parameter vector 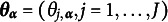 , given a membership 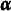 . Under their framework, GNPC method can be derived by taking 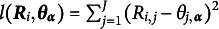 , which leads to minimizing the following loss function: (6)

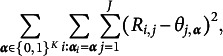

with respect to 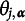 and 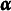 under constraint (1). To ensure identifiability, we impose the natural constraint 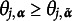 if 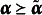 . Here 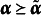 if 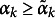 for all 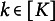 .

Note that given the membership 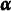 , the item parameter 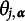 that minimizes the loss function (6) takes exactly the form of 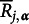 in (4) for all items and 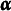 ’s. Inspired by this, as shown in Ma, de la Torre, et al. (2023), we can see that the solution 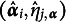 to the original GNPC estimation method in (5) is the same as the solution 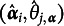 to (6) under constraint (1) and the following additional constraint: (7)

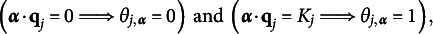

where the additional constraint (7) corresponds to the constraint (3) under the GNPC setting.

Following the above discussion, both the original GNPC method and the modified GNPC method can be formulated in a unified estimation framework (Ma, de la Torre, et al., 2023) of minimizing (6) under different constraints. In particular, since 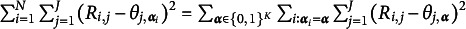 , we can rewrite (6) equivalently as the following loss function: (8)

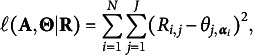

where minimizing the loss function (8) with respect to 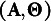 under the constraints (1) and (7) obtains the original GNPC estimators in Chiu and Köhn (2019a) and the modified GNPC estimators in Ma, de la Torre, et al. (2023) can be obtained by minimizing (8) under the constraint (1) only.

### 2.3 Limitations of existing consistency results for nonparametric CDMs

Existing theoretical research has offered valuable insights into the practical utility of nonparametric methods. It has been shown that the NPC estimators are statistically consistent for estimating subjects’ latent attributes under certain CDMs (Wang & Douglas, 2015). Similarly, the GNPC estimator’s ability to consistently classify subjects has been established (Chiu & Köhn, 2019a). However, current theoretical assurances for these nonparametric methods come with their own set of limitations.

A fundamental assumption for the NPC method to yield a statistically consistent estimator of 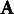 is that 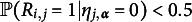 and 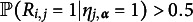 , where 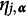 represents the binary ideal responses (either 0 or 1) under the considered model (Wang & Douglas, 2015). However, as previously pointed out, this binary ideal response becomes restrictive when working with more complex CDMs. The binary ideal response, limited to representing the complex latent attribute patterns of examinees through two states, could potentially oversimplify the actual complexity of the scenario. This limitation, in turn, constrains the practical application of the NPC method in instances where the underlying true model is more sophisticated. For instance, Chiu et al. (2018) provided an illustrative example highlighting this restriction, showing the possibility of misclassifications when the underlying true model is the saturated GDINA model.

Although the GNPC method addresses the oversimplification problem of the NPC method, a new restrictive assumption emerges in the existing theory for the GNPC method. Specifically, Theorem 1 in Chiu and Köhn (2019a) assumes initialization of the memberships 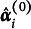 s that consistently estimates the ground truth in order to establish the consistency theory for GNPC. Similarly, Ma, de la Torre, et al. (2023) assumes the existence of a calibration dataset that provides consistent estimations 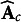 for the true latent class membership 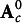 of the calibration subjects. Under these assumptions, 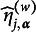 can be estimated using consistent membership estimations, which further support the consistency theory. The assumption concerning the existence of an initial set of consistent estimates or a calibration dataset may be restrictive and hard to satisfy in practice. To address this issue, we present new theoretical results demonstrating that the consistency of the GNPC method can be established without the need for a consistent initialization or a calibration dataset. These findings are detailed in the subsequent section.

## 3 Main results

Based on the unified framework of two GNPC methods outlined in Section 2, we will establish the theoretical properties of both the original GNPC method (Chiu & Köhn, 2019a) and the modified GNPC method (Ma, de la Torre, et al., 2023) under less stringent conditions. Regarding implementation, estimation algorithms for both the original and modified GNPC methods have been detailed in Chiu et al. (2018) and Ma, de la Torre, et al. (2023), respectively. We recommend using these well-established methods for estimation.

Before delving into the statistical behaviors of the aforementioned general nonparametric estimators, we outline the needed regularity conditions. Consider a model sequence indexed by 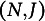 , where both *N* and *J* tend to infinity, while *K* is held constant. For clarity, let the true parameters generating the data be represented as 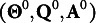 , and other true parameters are also denoted with superscript 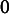 . Assumptions are made on these true parameters as follows.

Assumption 1. There exists 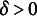 such that 

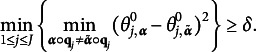

Assumption 2. There exist 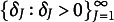 and a constant 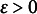 such that (9)

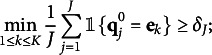


(10)

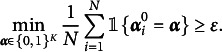

Assumption 1 serves as an identification condition for local latent classes at each item level, ensuring that the item parameters of different local latent classes, influenced by 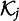 , are sufficiently distinct. The gap, denoted as 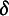 , measure the separation between latent classes, thereby quantifying the strength of the signals. In the finite-*J* regime, a 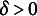 is required for studying identifiability (Gu & Xu, 2019, 2023; Köhn & Chiu, 2017; Xu & Shang, 2018). Assumption 2 pertains to the discrete structures of 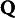 and 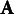 . Here, (10) implies that the 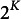 latent patterns are not too unevenly distributed in the sample. An equivalent requirement in random-effect latent class models is 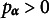 for all 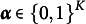 , where 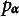 represents the population proportion of latent pattern 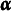 . For Assumption 2, within the finite-*J* regime, (9) is similar to the requirement that “ 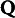 should contain an identity submatrix 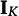 ” (Chen et al., 2015; Xu & Shang, 2018). However, as *J* approaches infinity, a finite number of submatrices 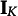 in 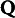 may not be sufficient to ensure estimability and consistency. Therefore, (9) necessitates that 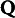 includes an increasing number of identity submatrices, 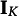 , as *J* grows. A similar assumption on 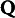 was made by Wang and Douglas (2015) when they were establishing the consistency of the NPC method. It is worth mentioning that the lower bound 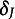 in (9) in Assumption 2 is allowed to decrease to zero as *J* goes to infinity.

In the following subsections, we study the consistency properties of the modified GNPC method with the constraint (1) and the original GNPC method with both constraints (1) and (7). As the modified GNPC method involves less constraints compared to the original GNPC method, for convenience, we first present results for the modified GNPC method in Section 3.1 and then for the original GNPC method in Section 3.2.

### 3.1 Consistency results for modified GNPC

In this section, we discuss the consistency results for the modified GNPC method. The following main theorem first validates the consistency of the modified GNPC method under the constraint (1) and provides a bound for its rate of convergence in recovering the latent attribute profiles. We use 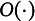 and 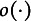 to denote the big-O and small-o notations, respectively, and 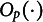 and 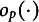 as their probability versions for convergence in probability.

Theorem 1 **(Consistency of modified GNPC method)**

Consider the 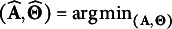 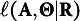 under the constraint (1). When 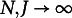 jointly, suppose 
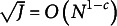
 for some small constant 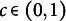 . Under Assumptions 1 and 2, the classification error rate is (11)

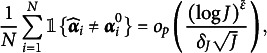

where for a small positive constant 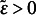 .

Theorem 1 bounds the error of the estimator 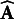 , which establishes the consistency of the latent attributes of the nonparametric method, and even allows the rate 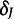 to go to zero. Theorem 1 also offers insight into the accuracy of estimating 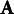 with finite samples and finite *J*. In particular, if 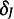 is a constant, then the finite sample error bound in (11) becomes 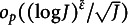 . Ignoring the 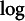 terms, the result shows that the classification error rate can be dominated by the order of 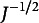 , indicating that a longer item set facilitates more accurate classification for the latent profiles of all subjects. Note the scaling condition that 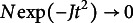 for any positive fixed 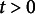 in Chiu and Köhn (2019a) and Wang and Douglas (2015) essentially requires the growth rate of *J* to be at least the order of 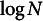 . In contrast, our scaling condition only assumes that the number of items goes jointly with *N* at a slower rate, which can be more easily satisfied.

The following corollary demonstrates that under certain conditions, the item parameters can be consistently estimated via the modified GNPC method as 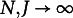 .

Theorem 2 **(Item Parameters Consistency)**

Under Assumptions 1 and 2 and the scaling conditions given in Theorem 1, we have the following uniform consistency result for all 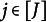 and 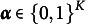 : 

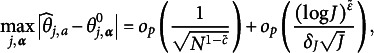

where 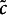 and 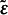 are small positive constants.

This theorem builds on the consistency result established in Theorem 1 to establish the uniform consistency in parameter estimation. The condition 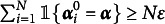 for all 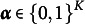 ensures that there are enough samples within each class to provide accurate estimates of item parameters. This is reflected in the first error term 
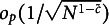
 , which achieves nearly optimal 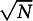 -consistency. Notably, the added 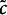 term arises due to the number of parameters going to infinity jointly with the sample size *N*, causing a slight deviation from the optimal error rate of 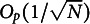 . The maximum deviation 
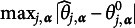
 is also influenced by the classification errors for the unknown latent attributes, which is shown in the second error term 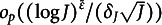 . In conclusion, the upper bound for the maximal error in estimating item parameters comprises a term that denotes nearly optimal 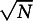 -consistency, accompanied by an additional term related to the errors in classifying the latent attributes. Our theory suggests that both the sample size and the test length need to be sufficiently large to ensure accurate estimation of the item parameters, given that the latent attributes of the subjects must also be estimated.

### 3.2 Consistency results for original GNPC

In this section, we discuss the consistency result of the original GNPC method. Since the original method adds an additional constraint (7) compared to the modified method, which causes some of the parameters 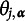 to be 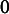 or 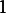 , additional notations are needed to characterize how this potential variation affects the consistency outcome. Denote (12)

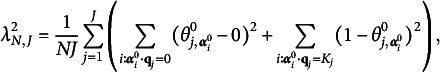

which represents the average squared distance between the true parameters and the associated zero/one values. To establish the consistency for the original GNPC method, an additional assumption is needed.

Assumption 3. For any 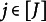 , we have 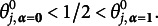

Assumption 3 plays a similar role to Assumption 1, as both measure the separation between different latent classes. While this appears to be a relatively mild condition and may seem similar to the one presented in Wang and Douglas (2015), as discussed in Section 2, it remains applicable to complex CDMs. The following theorem validate the consistency of the NPC method under the original GNPC setting, provides a similar bound as Theorem 1 for the misclassification rate.

Theorem 3 **(GNPC Consistency)**

Consider the 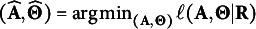 under the constraints (1) and (7). When 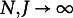 jointly, suppose 
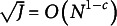
 for some small constant 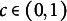 . Under Assumptions 2 and 3, the classification error rate is 

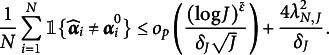

The classification error rate for the original GNPC method is slightly different from the result given in Theorem 1. An extra item 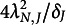 is added into the error rate. This additional term reflects the number of items that violate constraint (7), as (12) will be larger when there are more items with 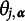 that is neither 0 nor 1. The impact of the additional error introduced by 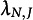 is further illustrated by Example 1 and the simulation studies in Section 4. The details of Theorem 3’s proof can be found in Section C of the Supplementary Material.

It is worth mentioning that without further regularity conditions, it might be challenging to avoid the additional error term 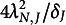 . In the existing consistency results for both the NPC and the modified GNPC methods (Chiu & Köhn, 2019a; Wang & Douglas, 2015), a crucial step involves ensuring that for each examinee *i*, the true attribute profile minimizes 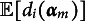 across all *m*. Here, 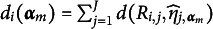 represents the distance functions used in the respective nonparametric methods. A similar approach is required in the proof of Theorem 1 for the modified GNPC method. If we denote 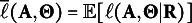 and 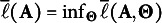 , then the true latent class profiles, 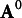 , are found to minimize 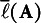 .

One challenge in establishing the consistency for the original GNPC method lies in the fact that, with the inclusion of the constraint (7), the true latent class profiles 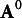 might not necessarily minimize 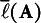 . Let 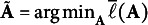, it can be intuitively understood that 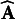 might approach 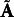 more closely than 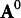 . Thus the additional error term originates from the discrepancy between 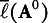 and 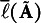 . Indeed, in the proof of Theorem 3, we employ the following upper bound to account for this deviation: (13)

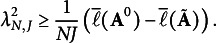



The above inequality in (13) is sharp up to a constant multiple of 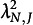 , below is an illustrative example.

Example 1. In this example, we assume that the number of sample size *N* is 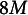 for some positive integer *M*, the number of items *J* is 4, and the dimension of latent attribute profiles *K* is also 4. We further assume that the four corresponding row vectors for the items in the Q-matrix are 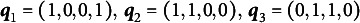 , and 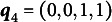 , where 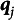 encodes the required latent attributes for the *j*th item. For the true latent attribute profiles of the 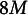 samples, it is assumed that 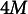 samples exhibit latent attribute profile 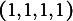 , while the remaining 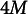 display the profile 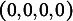 . It is noteworthy that all parameters in the original GNPC method will be treated as exactly zero or one under the true latent attribute profiles 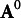 in this example, as stipulated by the constraint (7). The last assumption in this example is that there exists some 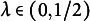 such that 
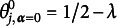
 and 
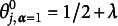
 . Under these assumptions, the expected loss under the true latent attribute profiles satisfies (14)

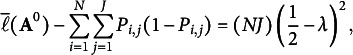

where 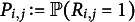 are true item response parameters, independent of the estimation process. The derivation of (14) is detailed in Section D of the Supplementary Material. To demonstrate the sharpness of inequality (13), we construct an alternative set of latent attribute profiles, denoted as 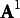 . This set contains 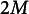 samples of 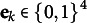 for each 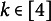 , where each 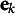 only contains the *k*th latent attribute. For instance, 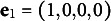 , 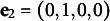 , and so on. There is a correspondence between the true latent profiles 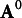 and the constructed 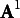 . Specifically, for the 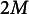 samples assigned to 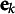 within 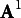 , the true latent attribute profiles are equally divided, with half being 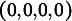 and the other half 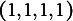 . Hence, the expected loss under the constructed latent attribute profiles fulfills (15)

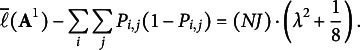

The derivation of (15) is detailed in Section D of the Supplementary Material. Thus, we have 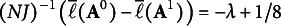 . Note that in this example 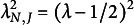 . If 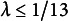 , then one can easily verify that 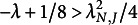 , and therefore, in this case, we deduce that 

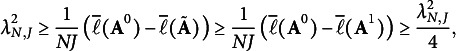

which implies the order 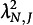 in the inequality in (13) is sharp. The details of the proof can be found in Section D of the Supplementary Material.

The magnitude of the additional classification error term arising from the aforementioned discrepancy is of the order 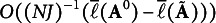 . As demonstrated in Example 1, 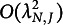 provides a tight estimation of the order of the discrepancy 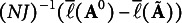 . Therefore, the additional error term 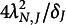 in Theorem 3 may not be significantly reducible.

## 4 Simulation study

In this section, we conduct a comprehensive simulation study to illustrate our theoretical findings of both the original GNPC (Chiu et al., 2018) and the modified GNPC (Ma, de la Torre, et al., 2023). Note that in the existing literature (Chiu et al., 2018; Ma, de la Torre, et al., 2023), various numerical studies have already demonstrated the effectiveness of both the original GNPC method and the modified GNPC method in small sample settings. Therefore, our focus here primarily lies on scenarios where both the sample size and test length are relatively large to illustrate our theoretical results.

For the data-generating process, followed by the simulation design of Chiu et al. (2018) and Ma, de la Torre, et al. (2023), we consider two settings: (1) items are simulated using the DINA model, and (2) items are simulated from GDINA model, as detailed in Section 2. The manipulated conditions include: the sample size 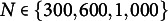 ; the test length 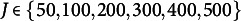 ; the number of latent attributes 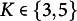 . For 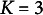 , the Q-matrix is constructed with two identity 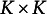 submatrices, and the remaining items are generated uniformly from all possible non-zero patterns. It is worth mentioning that this generating process adheres to (9) in Assumption 2. For the case of 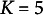 , the Q-matrix is restricted to contain items that measure up to three attributes and constructed the same way as that for 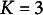 . For the data conforming to the DINA model, we simulate 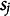 and 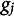 independently from a uniform distribution 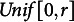 with 
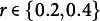
 . For data generated under the GDINA model, the item parameters are simulated following the framework outlined in Chiu et al. (2018) as follows. For any item *j*, let 
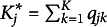
 be the number of required attributes of item *j*, where 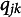 is the 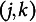 th entry in the Q-matrix. Without loss of generality, we assume that these attributes with 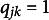 are the first 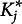 attributes. For instance, if 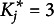 , that is, item *j* requires three attributes, we then denote the possible proficiency classes as 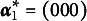 , 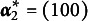 , 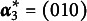 , 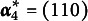 , 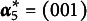 , 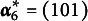 , 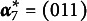 , and 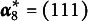 . The item parameters for item *j* are specified by the probabilities of making the correct responses for all 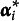 with 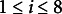 . If 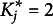 , we only need to specify the probabilities for 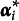 with 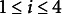 since the remaining attributes are irrelevant for distinguishing among the proficiency classes, and if 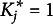 , we only need to specify the probabilities for 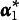 and 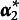 (Chiu et al., 2018). Analogous to the data generation process under the DINA model, we simulate two noise levels under the GDINA model as in Ma, de la Torre, et al. (2023), with item parameters provided in Table 1 for small noises and Table 2 for large noises, respectively. Note that Table 1 contains seven rows, while Table 2 contains six, each row representing a distinct set of item parameters. For each noise level, the set of item parameters for each item *j* is sampled randomly from those rows with 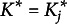 in each table.

**Table 1:** Item response parameters for GDINA with small noises, where 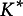 denotes the number of required attributes of a considered item

	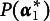	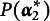	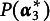	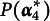	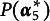	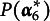	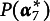	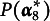
	0.2	0.9						
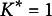	0.1	0.8						
	0.1	0.9						
	0.2	0.5	0.4	0.9				
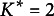	0.1	0.3	0.5	0.9				
	0.1	0.2	0.6	0.8				
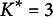	0.1	0.2	0.3	0.4	0.4	0.5	0.7	0.9

**Table 2:** Item response parameters for GDINA with large noises, where 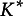 denotes the number of required attributes of a considered item

	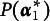	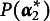	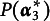	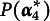	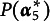	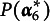	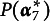	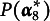
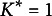	0.3	0.7						
	0.3	0.8						
	0.3	0.4	0.7	0.8				
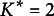	0.3	0.4	0.6	0.7				
	0.2	0.3	0.6	0.7				
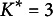	0.2	0.3	0.3	0.4	0.4	0.5	0.6	0.7

For the latent attribute patterns, they are generated using either a uniform setting, where each proficiency class is drawn with a uniform probability of 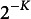 , or a multivariate normal threshold model as described by Chiu et al. (2018). In this model, each subject’s attribute profile is linked to a latent continuous ability vector 
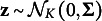
 . The diagonal elements of 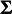 are fixed at 1, while the off-diagonal elements are set to 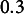 for a low-correlation scenario and 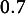 for a high correlation scenario. The attribute profile is then derived from 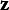 by applying a truncation process as follows: 

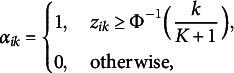

where 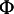 is the cumulative distribution function of the standard normal distribution.

To illustrate our theoretical results, we compute the pattern-wise agreement rate (PAR): 

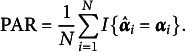

We apply the original GNPC and modified GNPC estimation methods under all manipulated scenarios. Following the algorithms proposed by Chiu et al. (2018) and Ma, de la Torre, et al. (2023), both methods are initialized using latent attributes estimated with the NPC method for computational efficiency. In each scenario, we conduct 100 replications and calculate the mean value of the PARs. The iteration process is terminated when 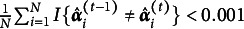 or exceeding the maximal number of iterations set as 500. Additionally, we conducted simulations in these scenarios where both methods are initialized with random latent attributes, resulting in estimation errors similar to those obtained with NPC initialization. Details and results are provided in Section E of the Supplementary Material.

**Figure 1:**
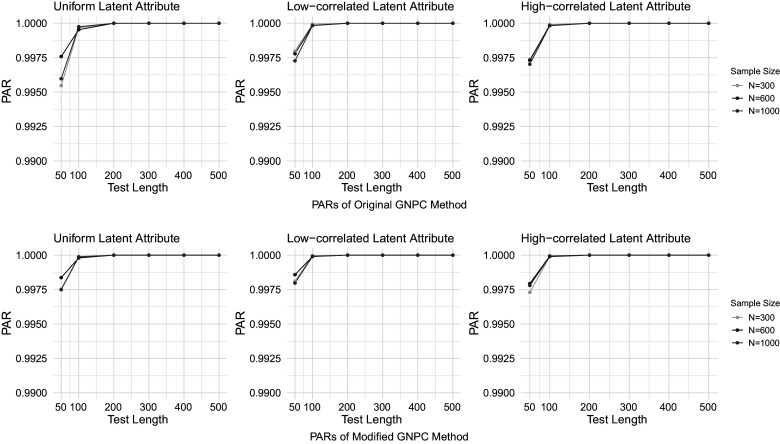
PARs when the data are generated using the DINA model with 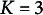 and 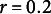 .

**Figure 2:**
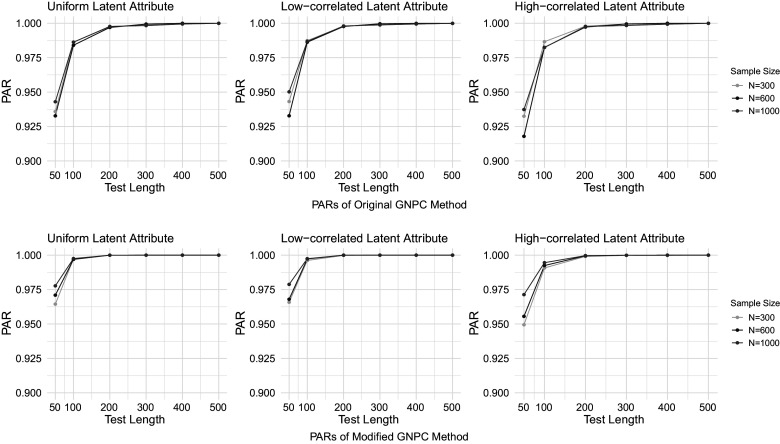
PARs when the data are generated using the DINA model with 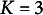 and 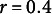 .

**Figure 3:**
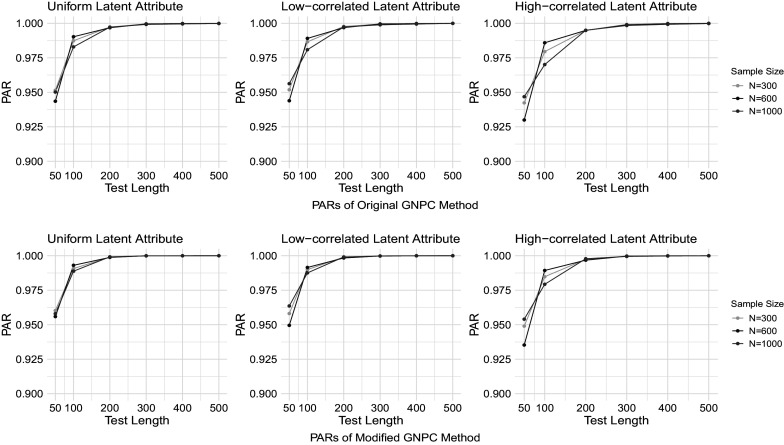
PARs when the data are generated using the DINA model with 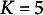 and 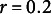 .

**Figure 4:**
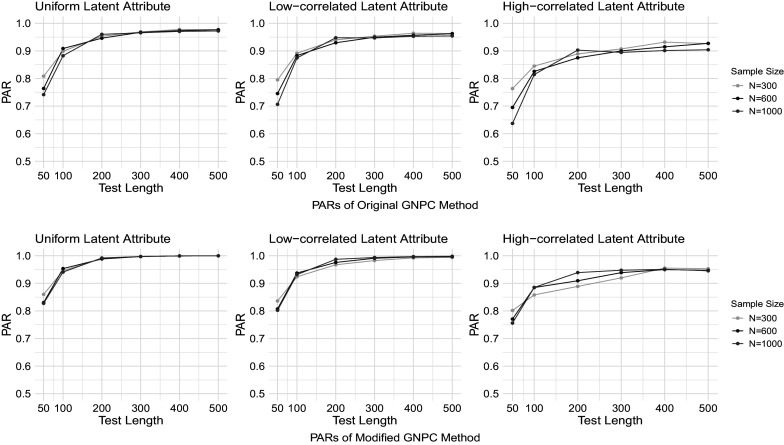
PARs when the data are generated using the DINA model with 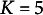 and 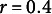 .

**Figure 5:**
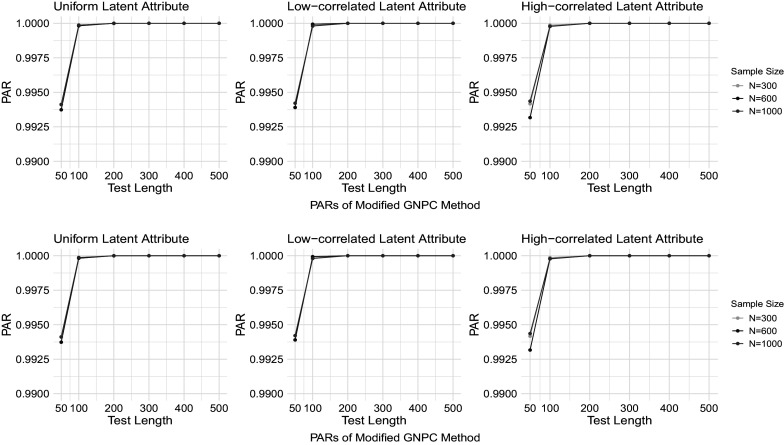
PARs when the data are generated using the GDINA model with small noises and 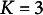 .

**Figure 6:**
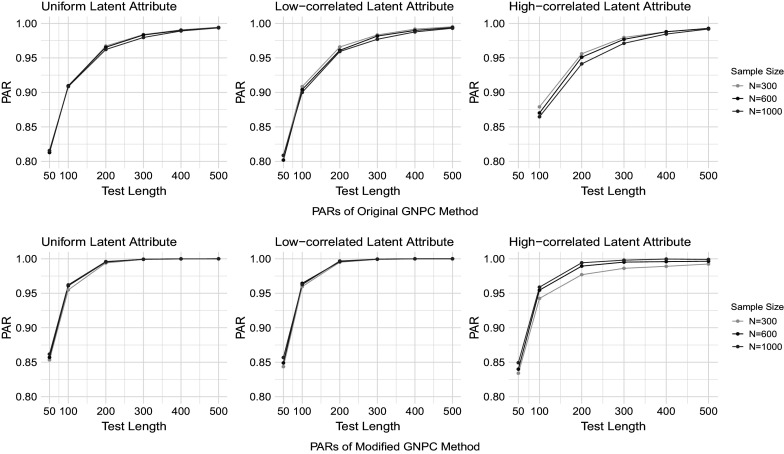
PARs when the data are generated using the GDINA model with large noises and 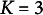 .

**Figure 7:**
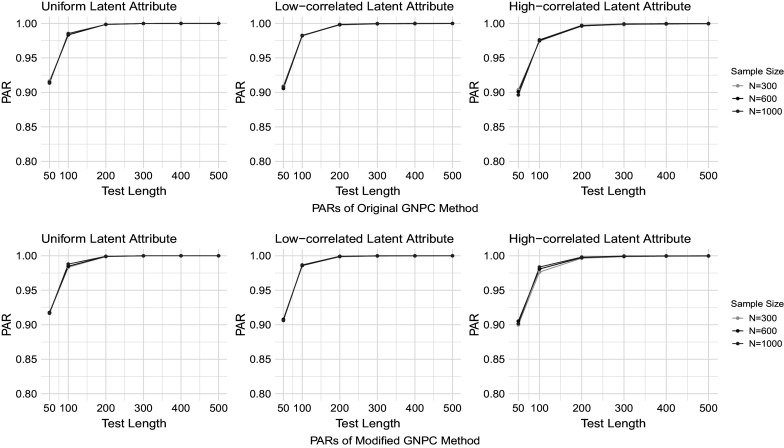
PARs when the data are generated using the GDINA model with small noises and 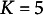 .

**Figure 8:**
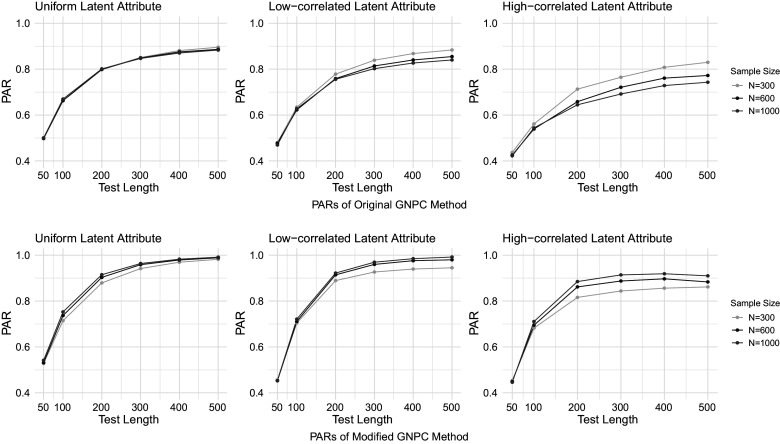
PARs when the data are generated using the GDINA model with large noises and 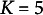 .

Figures 1–4 present the PAR results when the data are generated under the DINA model, and the PAR results under the GDINA model are shown in Figures 5–8. In each figure, the upper panel presents the estimation results using the original GNPC method, while the lower panel displays the results using the modified GNPC method. From left to right, the subfigures in each row illustrate the estimations for latent attributes, simulated under three different settings: uniform, low correlation setting, and high correlation setting.

In general, both the original and modified GNPC methods perform well under all the model settings. The modified GNPC method exhibits a slight edge in more complex scenarios, as demonstrated in Figures 4 and 8. A consistent trend across all figures is that as the test length *J* increases, the PARs improve, supporting our theory that the upper bound for classification error decreases with *J*. When the data are simulated from the GDINA models, there is a slight increase in classification errors for both methods compared to those generated using the DINA models. In addition, comparisons between figures with lower noise levels (Figures 1, 3, 5, and 7) and those with higher ones (Figures 2, 4, 6, and 8) reveal lower classification errors with decreased noise. In particular, Figures 1 and 5 show nearly perfect classification results under low noise and 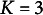 settings. Moreover, increasing the number of latent attributes typically results in less precise estimation, as evidenced by the comparisons between the settings of 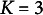 (Figures 1, 2, 5, and 6) and 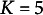 (Figures 3, 4, 7, and 8). Within each figure, a slight decrease in PARs is observed when the latent attributes exhibit a higher correlation. When the data are simulated under larger noises and more attributes (Figure 4 and 8), PARs from the original GNPC method appear not converging to 1 even when the sample size is 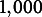 and test length is 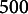 . This is likely attributable to the additional error term related to 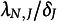 in Theorem 3. Notably, 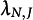 in (12) can become large when a significant proportion of 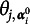 fails to satisfy the constraint (7).

## 5 Discussion

In this work, we revise the consistency results for the GNPC method, originally offered in Chiu and Köhn (2019a), under relaxed and more practical assumptions. We deliver finite sample error bounds for the considered two versions of the GNPC method. These bounds not only guarantee asymptotic consistency in estimating the latent profiles of subjects but also offer insights into the precision of these estimates in small sample situations. Furthermore, we derive uniform convergence of item response parameters 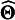 for the modified GNPC method. Notably, all of these advancements are achieved without the requirement for a calibration dataset.

The findings in this study open up several possibilities for future exploration. Using the consistency and finite sample error bounds established for estimating the discrete latent structure 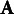 , future work can examine statistical inference on CDMs with a large number of test items and latent attributes. Additionally, it is important to note that in practical situations, the Q-matrix may not always be readily available. Various estimation techniques have been proposed in the literature (Chen et al., 2015, 2018; Gu & Xu, 2023; Köhn et al., 2024; Li et al., 2022; Liu et al., 2012; Ma, Ouyang, et al., 2023; Xu & Shang, 2018). This leads to a potential future direction of developing theories and computational methods for CDMs estimation with an unknown Q-matrix within the nonparametric framework.

## Supporting information

Cui et al. supplementary material Cui et al. supplementary material

## Supplementary material

The supplementary material for this article can be found at https://doi.org/10.1017/psy.2025.9.
