# Supplementary material for: Consistency Theory of General Nonparametric Classification Methods in Cognitive Diagnosis
Source: Psychometrika. 2025 Mar 17;90(3):1136–52. doi: 10.1017/psy.2025.9 (PMC12483721; doi:10.1017/psy.2025.9)
Supplement: Cui et al. supplementary material [file S0033312325000092sup001.pdf]

Supplementary Material for "Consistency Theory of General Nonparametric Classification Methods in Cognitive Diagnosis"

Chengyu Cui[1], Yanlong Liu[2]*, and Gongjun Xu[1]

University of Michigan[1] and University of Chicago[2]

In this supplementary material, we provide proofs for the theoretical results of our study. Section A provides notations that are used throughout the Supplementary Material. The proofs for Theorem 1 and Theorem 2 are presented in Section B. Section C presents the proofs for Theorem 3 and Section D provides details of derivations in Example 1. Section E presents additional simulation results.

A Preliminaries

Motivated by the constraint (1), we introduce the concept of a "local" latent class at the item level. Considering item j with Q-vector $\boldsymbol{q}_j$, the constraint (1) divides the collection of latent attribute profiles $\boldsymbol{\alpha}$, which is $\{0,1\}^K$, based on an equivalence relationship where $\boldsymbol{\alpha} \sim_j \tilde{\boldsymbol{\alpha}}$ is defined by $\boldsymbol{\alpha} \circ \boldsymbol{q}_j = \tilde{\boldsymbol{\alpha}} \circ \boldsymbol{q}_j$, here the subscript $\sim_j$ emphasizes that the equivalence relationship is determined by the j-th item $\boldsymbol{q}_j$. On this basis, we introduce a function $\xi : \{0,1\}^K \times \{0,1\}^K \to \mathbb{N}$ where $\xi(\boldsymbol{q}_j, \boldsymbol{\alpha}) = \xi(\boldsymbol{q}_j, \tilde{\boldsymbol{\alpha}})$ is equivalent to $\boldsymbol{\alpha} \circ \boldsymbol{q}_j = \tilde{\boldsymbol{\alpha}} \circ \boldsymbol{q}_j$. This function assigns numbers to these equivalence classes induced by item j based on

*Cui and Liu are co-first authors

some specific rules. In the following context, we call $\xi(\boldsymbol{q}_j^0, \boldsymbol{\alpha})$ the local latent class of $\boldsymbol{\alpha}$ induced by item j. It is straightforward to verify that the number of the local latent classes induced by item j, denoted by $|\xi(\boldsymbol{q}_j, \{0,1\}^K)|$, equals $L_j = 2^{K_j}$. Here, $K_j = \sum_{k=1}^K q_{j,k}^0$ represents the number of the required latent attributes for item j. Consequently, we let the range of the function ξ satisfies $\xi(\boldsymbol{q}_j, \{0,1\}^K) = [L_j] := \{1, \ldots, L_j\}$. Since the local latent classes are identified up to permutations on $[L_j]$ due to their categorical nature, the mapping rules between $\xi(\boldsymbol{q}_j, \{0,1\}^K)$ and $[L_j]$ do not need to be completely specified in the discussion of the modified GNPC method. However, as indicated by the constraint (7) and Assumption 3, the two latent attribute profiles $(0, \ldots, 0)$ and $(1, \ldots, 1)$ induce the deviation between the modified GNPC and the original GNPC methods. Thus, we specify $\xi(\boldsymbol{q}_j, \mathbf{0}) = 1$ and $\xi(\boldsymbol{q}_j, \mathbf{1}) = L_j$ for any item j in the discussion of the original GNPC method.

For brevity, we use a general notation $\mathbf{Z} = (z_{i,j})$ to denote the collection of the local latent classes for all items $j \in [J]$ and subjects $i \in [N]$, where $z_{i,j}$ represents $\xi(\boldsymbol{q}_j^0, \boldsymbol{\alpha}_i)$. Given that $\xi(\boldsymbol{q}_j^0, \boldsymbol{\alpha}) = \xi(\boldsymbol{q}_j^0, \tilde{\boldsymbol{\alpha}})$ implies $\theta_{j,\boldsymbol{\alpha}} = \theta_{j,\tilde{\boldsymbol{\alpha}}}$ by the definition of ξ, we express $\theta_{j,\boldsymbol{\alpha}_i}$ as $\theta_{j,z_{i,j}}$ to directly incorporate the constraint (1) into the loss function (8). For further notational simplicity, we may sometimes write $\theta_{j,z_{i,j}}$ simply as θ_{j,z_i}. Consequently, we define

$$P_{i,j} = \mathbb{P}(R_{i,j} = 1) = \theta^0_{j,z_i^0}. \tag{A.1}$$

Then the loss function (8) can be rewritten as

$$\ell(\mathbf{A}, \boldsymbol{\Theta}|\mathbf{R}) = \sum_{i=1}^N \sum_{j=1}^J (R_{i,j} - \theta_{j,z_i})^2. \tag{A.2}$$

Observe that $R_{i,j}^2 = R_{i,j}$, and $\mathbb{E}[R_{i,j}] = P_{i,j}$, we denote the expectation of the above $\ell(\mathbf{A}, \boldsymbol{\Theta}|\mathbf{R})$ by

$$\bar{\ell}(\mathbf{A}, \boldsymbol{\Theta}) := \mathbb{E}[\ell(\mathbf{A}, \boldsymbol{\Theta}|\mathbf{R})] = \sum_{i=1}^N \sum_{j=1}^J (P_{i,j} - \theta_{j,z_i})^2 + \sum_{i=1}^N \sum_{j=1}^J P_{i,j}(1 - P_{i,j}). \tag{A.3}$$

Note $\mathbf{Z} = (z_{i,j})$ is only determined by $\mathbf{A}$ since $\mathbf{Q}^0$ is known. In the subsequent context, the quantities that are determined by the latent attribute profiles $\mathbf{A}$ are sometimes denoted

with a superscript $\mathbf{A}$ to emphasize their relationships with $\mathbf{A}$. Given an arbitrary $\mathbf{A}$, denote

$$\ell(\mathbf{A}) = \inf_{\boldsymbol{\Theta}} \ell(\mathbf{A}, \boldsymbol{\Theta}|\mathbf{R}) = \ell(\mathbf{A}, \widehat{\boldsymbol{\Theta}}^{(\mathbf{A})}|\mathbf{R}); \tag{A.4}$$

$$\overline{\ell}(\mathbf{A}) = \inf_{\boldsymbol{\Theta}} \overline{\ell}(\mathbf{A}, \boldsymbol{\Theta}) = \overline{\ell}(\mathbf{A}, \overline{\boldsymbol{\Theta}}^{(\mathbf{A})}), \tag{A.5}$$

where $\widehat{\boldsymbol{\Theta}}^{(\mathbf{A})} := \arg\min_{\boldsymbol{\Theta}} \ell(\mathbf{A}, \boldsymbol{\Theta}|\mathbf{R})$ and $\overline{\boldsymbol{\Theta}}^{(\mathbf{A})} := \arg\min_{\boldsymbol{\Theta}} \overline{\ell}(\mathbf{A}, \boldsymbol{\Theta})$. Then under any realization of $\mathbf{A}$, the following equations hold for any local latent class $a \in [L_j]$:

$$\widehat{\theta}_{j,a}^{(\mathbf{A})} = \frac{\sum_{i=1}^{N} \mathbb{1}\{z_{i,j}^{(\mathbf{A})} = a\} R_{i,j}}{\sum_{i=1}^{N} \mathbb{1}\{z_{i,j}^{(\mathbf{A})} = a\}}, \quad \overline{\theta}_{j,a}^{(\mathbf{A})} = \frac{\sum_{i=1}^{N} \mathbb{1}\{z_{i,j}^{(\mathbf{A})} = a\} P_{i,j}}{\sum_{i=1}^{N} \mathbb{1}\{z_{i,j}^{(\mathbf{A})} = a\}}. \tag{A.6}$$

To derive (A.6), note the sum $\sum_{j=1}^{J} \sum_{i=1}^{N} (R_{i,j} - \theta_{j,z_i})^2$ equals the sum $\sum_{j=1}^{J} \sum_{a=1}^{L_j} \sum_{z_i=a} (R_{i,j} - \theta_{j,a})^2$. When estimating $\widehat{\theta}_{j,a}$, we focus on minimizing the term $\sum_{z_i=a} (R_{i,j} - \theta_{j,a})^2$. For $\overline{\theta}_{j,a}$, note that $\sum_i \sum_j P_{i,j}(1 - P_{i,j})$ is independent of the estimation processes. A useful observation is that, by plugging $\mathbb{E}[R_{i,j}] = P_{i,j}$ into (A.6), we can find that $\mathbb{E}[\widehat{\theta}_{j,a}] = \overline{\theta}_{j,a}$ holds for any (j, a).

B Proofs of Theorem 1 and Theorem 2

We first outline the main steps of the proof of Theorem 1 as follows and then proceed one by one.

B.1 Outline of the first half of the proof

Step 1: Express $\overline{\ell}(\mathbf{A}) - \ell(\mathbf{A})$ by $\sum_{j=1}^{J} \sum_{a=1}^{L_j} n_{j,a} (\widehat{\theta}_{j,a} - \overline{\theta}_{j,a})^2 + \mathbb{E}[X] - X$, where $X := \sum_i \sum_j R_{i,j}(1 - 2\overline{\theta}_{j,z_i})$ depending on $\mathbf{R}$ and $\overline{\boldsymbol{\Theta}}^{(\mathbf{A})}$ under $\mathbf{A}$, and $n_{j,a} := \sum_{i=1}^{N} \mathbb{1}\{z_{i,j}^{(\mathbf{A})} = a\}$.

Step 2: Bound $\sum_j \sum_a n_{j,a} (\widehat{\theta}_{j,a} - \overline{\theta}_{j,a})^2$ and $|X - \mathbb{E}[X]|$ separately to obtain a uniform convergence rate $\sup_{\mathbf{A}} \left|\overline{\ell}(\mathbf{A}) - \ell(\mathbf{A})\right| = o_p(\delta_{N,J})$

Step 3: By noting the closed form of $\overline{\ell}(\mathbf{A}) - \overline{\ell}(\mathbf{A}^0) = \sum_i \sum_j (P_{i,j} - \overline{\theta}_{j,z_i}^{(\mathbf{A})})^2$ for any $\mathbf{A}$, we deduce that $\overline{\ell}(\mathbf{A}) \geq \overline{\ell}(\mathbf{A}^0)$ holds for all $\mathbf{A}$. Based on the definition of $\widehat{\mathbf{A}}$, it follows that $0 \leq \overline{\ell}(\widehat{\mathbf{A}}) - \overline{\ell}(\mathbf{A}^0) \leq 2 \sup_{\mathbf{A}} |\overline{\ell}(\mathbf{A}) - \ell(\mathbf{A})| = o_p(\delta_{N,J})$, which controls the deviation $\overline{\ell}(\widehat{\mathbf{A}}) - \overline{\ell}(\mathbf{A}^0)$.

In some classical statistical inference contexts, consistency results for the parameters of interest are typically established through the uniform convergence of random functions associated with these parameters. For instance, if $\sup_{\boldsymbol{\theta}\in\boldsymbol{\Theta}} \left|\widehat{\ell}(\boldsymbol{\theta}) - \ell(\boldsymbol{\theta})\right| \xrightarrow{\mathbb{P}} 0$, and if we further assume that ℓ has a unique minimum $\tilde{\boldsymbol{\theta}}$ on $\boldsymbol{\Theta}$, then $\arg\min_{\boldsymbol{\Theta}} \widehat{\ell}(\boldsymbol{\theta}) =: \widehat{\boldsymbol{\theta}} \xrightarrow{\mathbb{P}} \tilde{\boldsymbol{\theta}}$ under some regularity conditions. The regularity conditions might vary across different settings. Consider $\mathbf{A}$ as the parameter to be estimated, the primary aim in the first three steps is to show that $\mathbf{A}$ minimizes the expected loss and establish a uniform convergence result for the random loss function of $\mathbf{A}$.

B.2 Outline of the second half of the proof

Step 4: Define $N_{a,b}^j = \sum_{i=1}^N \mathbb{1}\{z_{i,j}^0 = a\}\mathbb{1}\{\widehat{z}_{i,j} = b\}$, $a, b \in [L_j]$ to represent the samples with the wrong local latent class assignments. Derive some upper bounds for the quantities based on $N_{a,b}^j$ by using $\overline{\ell}(\widehat{\mathbf{A}}) - \overline{\ell}(\mathbf{A}^0)$ with the help of the identification assumptions.

Step 5: Bound the $\sum_{i=1}^N \mathbb{1}\{\widehat{\boldsymbol{\alpha}}_i \neq \boldsymbol{\alpha}^0\}$ by using the quantities based on $N_{a,b}^j$ with the help of the discrete structure of the Q-matrix, then obtain the desired classification error rate.

Assumption 1 and Assumption 2 are the regularity conditions for achieving clustering consistency based on the uniform convergence results established in the first half of the proof. We will make more comments about the assumptions in the later proofs.

B.3 First Half of the Proof of Theorem 1

Step 1. The idea of decomposing $\overline{\ell}(\mathbf{A}) - \ell(\mathbf{A})$ is to consider

$$\ell(R_{i,j}, \widehat{\theta}_{j,z_i}) - \mathbb{E}[\ell(R_{i,j}, \overline{\theta}_{j,z_i})] = \Big(\ell(R_{i,j}, \widehat{\theta}_{j,z_i}) - \ell(R_{i,j}, \overline{\theta}_{j,z_i})\Big) + \big(\ell(R_{i,j}, \overline{\theta}_{j,z_i}) - \mathbb{E}[\ell(R_{i,j}, \overline{\theta}_{j,z_i})]\big)\,.$$

The variability in the first term of the right-hand side mainly from the fluctuation in $\left|\widehat{\theta}_{j,a} - \overline{\theta}_{j,a}\right|$, while the randomness in the second term is due to the stochastic nature of $R_{i,j}$.

Lemma 1. *Let $(R_{i,j}; 1 \le i \le N, 1 \le j \le J)$ denote independent Bernoulli trials with parameters $(P_{i,j}; 1 \le i \le N, 1 \le j \le J)$. Under a general latent class model, given an*

arbitrary latent attribute profiles $\mathbf{A}$*, there is*

$$
\begin{aligned}
&\inf_{\mathbf{\Theta}} \mathbb{E}[\ell(\mathbf{A}, \mathbf{\Theta}|\mathbf{R})] - \inf_{\mathbf{\Theta}} \ell(\mathbf{A}, \mathbf{\Theta}|\mathbf{R}) \\
=&\sum_{j=1}^{J} \sum_{a=1}^{L_j} n_{j,a}(\widehat{\theta}_{j,a} - \overline{\theta}_{j,a})^2 + \sum_{i=1}^{N} \sum_{j=1}^{J} (P_{i,j} - R_{i,j})(1 - 2\overline{\theta}_{j,z_i}) \\
=&\sum_{j=1}^{J} \sum_{a=1}^{L_j} n_{j,a}(\widehat{\theta}_{j,a} - \overline{\theta}_{j,a})^2 + \mathbb{E}[X] - X, \qquad \text{(A.7)}
\end{aligned}
$$

where $X = \sum_{j=1}^{J} \sum_{i=1}^{N} R_{i,j}(1 - 2\overline{\theta}_{j,z_i})$ *is a random variable depending on* $\mathbf{A}$ *and* L_j *denotes the number of the distinct local latent classes induced by* $\boldsymbol{q}_j$ *for item* j*.*

Proof. Note $\overline{\ell}(\mathbf{A}) = \sum_i \sum_j (P_{i,j} - \overline{\theta}_{j,z_i})^2 + \sum_i \sum_j P_{i,j}(1 - P_{i,j})$, then

$$
\begin{aligned}
&\overline{\ell}(\mathbf{A}) - \ell(\mathbf{A}) \\
=&\sum_i \sum_j \Big((P_{i,j} - \overline{\theta}_{j,z_i})^2 - (R_{i,j} - \widehat{\theta}_{j,z_i})^2 \Big) + \sum_i \sum_j P_{i,j}(1 - P_{i,j}) \\
=&\sum_i \sum_j \Big((R_{i,j} - \overline{\theta}_{j,z_i})^2 - (R_{i,j} - \widehat{\theta}_{j,z_i})^2 \Big) \\
&+ \sum_i \sum_j \big((P_{i,j} - \overline{\theta}_{j,z_i})^2 - (R_{i,j} - \overline{\theta}_{j,z_i})^2 \big) + \sum_{i=1}^{N} \sum_{j=1}^{J} P_{i,j}(1 - P_{i,j}) \\
=&\sum_i \sum_j \Big((R_{i,j} - \overline{\theta}_{j,z_i})^2 - (R_{i,j} - \widehat{\theta}_{j,z_i})^2 \Big) + \sum_i \sum_j (P_{i,j} - R_{i,j})(1 - 2\overline{\theta}_{j,z_i}).
\end{aligned}
$$

The last equality holds since $R_{i,j}^2 = R_{i,j}$. Given a fixed $\mathbf{A}$, (A.6) implies that $\sum_{z_{i,j}=a} R_{i,j} =$

$n_{j,a}\widehat{\theta}_{j,a}$, $\sum_{z_{i,j}=a} P_{i,j} = n_{j,a}\overline{\theta}_{j,a}$, then

$$\begin{aligned}
&\sum_i \sum_j \left((R_{i,j} - \overline{\theta}_{j,z_i})^2 - (R_{i,j} - \widehat{\theta}_{j,z_i})^2\right) \\
=&\sum_{j=1}^{J}\sum_{a=1}^{L_j}\sum_{z_i=a} \left((R_{i,j} - \overline{\theta}_{j,a})^2 - (R_{i,j} - \widehat{\theta}_{j,a})^2\right) \\
=&\sum_{j=1}^{J}\sum_{a=1}^{L_j}\sum_{z_i=a} \left(2R_{i,j}\widehat{\theta}_{j,a} - 2R_{i,j}\overline{\theta}_{j,a} + \overline{\theta}_{j,a}^2 - \widehat{\theta}_{j,a}^2\right) \\
=&\sum_{j=1}^{J}\sum_{a=1}^{L_j} \left(2n_{j,a}\widehat{\theta}_{j,a}^2 - 2n_{j,a}\widehat{\theta}_{j,a}\overline{\theta}_{j,a} + n_{j,a}\overline{\theta}_{j,a}^2 - n_{j,a}\widehat{\theta}_{j,a}^2\right) \\
=&\sum_{j=1}^{J}\sum_{a=1}^{L_j} n_{j,a}(\widehat{\theta}_{j,a} - \overline{\theta}_{j,a})^2. \qquad \text{(A.8)}
\end{aligned}$$

This completes the proof of the lemma. □

Step 2. In this step we bound $\sum_j \sum_a n_{j,a}(\widehat{\theta}_{j,a} - \overline{\theta}_{j,a})^2$ and $|X - \mathbb{E}[X]|$ separately, for bounding the first term, we have the following lemma.

Lemma 2. *The following event happens with probability at least* $1 - \delta$*,*

$$\max_{\mathbf{A}} \left\{ \sum_{j=1}^{J}\sum_{a=1}^{L_j} n_{j,a}(\widehat{\theta}_{j,a} - \overline{\theta}_{j,a})^2 \right\} < \frac{1}{2}\left(N \log 2^K + J2^K \log\left(\frac{N}{2^K} + 1\right) - \log\delta\right).$$

Proof. Under any realization of $\mathbf{A}$, each $\widehat{\theta}_{j,a}$ is an average of $n_{j,a}$ independent Bernoulli random variables $r_{1,j}, \ldots, r_{N,j}$ with mean $\overline{\theta}_{j,a}$. By applying the Hoeffding inequality, we have

$$\mathbb{P}(\widehat{\theta}_{j,a} \geq \overline{\theta}_{j,a} + t) \leq \exp(-2n_{j,a}t^2), \quad \mathbb{P}(\widehat{\theta}_{j,a} \leq \overline{\theta}_{j,a} - t) \leq \exp(-2n_{j,a}t^2). \qquad \text{(A.9)}$$

Note that given a fixed $\mathbf{A}$, each $\widehat{\theta}_{j,a}$ can take values only in the finite set $\{0, 1/n_{j,a}, 2/n_{j,a}, \ldots, 1\}$ of cardinality $n_{j,a} + 1$. We denote this range of $\widehat{\theta}_{j,a}$ by $\widehat{\Theta}^{j,a}$ and denote the range of the matrix $\widehat{\mathbf{\Theta}} = (\theta_{j,a})$ by $\widehat{\Theta}$. Then $\mathbb{P}(\widehat{\theta}_{j,a} = v) \leq \exp(-2n_a(v - \overline{\theta}_{j,a})^2)$ for any $v \in \widehat{\Theta}^{j,a}$. Since for each of the $J \times 2^K$ entries in $\widehat{\mathbf{\Theta}}$, $\widehat{\theta}_{j,a}$ can independently take on $n_{j,a} + 1$ different values, there is $\left|\widehat{\Theta}\right| = \prod_j \prod_{a=1}^{L_j}(n_{j,a} + 1)$ with constraint $\sum_{a=1}^{L_j} n_{j,a} = N$. Since $L_j = 2^{K_j} \leq 2^K$, we have $\prod_{a=1}^{L_j}(n_{j,a} + 1) \leq (1 + N/2^K)^{2^K}$. Denote $\widehat{\Theta}_\epsilon = \{\widehat{\mathbf{\Theta}} \in \widehat{\Theta} : \sum_j \sum_a n_{j,a}(\tilde{\theta}_{j,a} - \overline{\theta}_{j,a})^2 \geq \epsilon\}$,

then $\widehat{\Theta}_\epsilon \subseteq \widehat{\Theta}$, and

$$\begin{aligned}
\mathbb{P}\left(\sum_j \sum_{a=1}^{L_j} n_{j,a}(\widehat{\theta}_{j,a} - \overline{\theta}_{j,a})^2 \geq \epsilon\right) &= \sum_{\tilde{\boldsymbol{\Theta}} \in \widehat{\Theta}_\epsilon} \mathbb{P}\left(\widehat{\boldsymbol{\Theta}} = \tilde{\boldsymbol{\Theta}}\right) \\
&\leq \sum_{\tilde{\boldsymbol{\Theta}} \in \widehat{\Theta}_\epsilon} \prod_j \prod_a \exp\left(-2n_{j,a}(\tilde{\theta}_{j,a} - \overline{\theta}_{j,a})^2\right) \\
&= \sum_{\tilde{\boldsymbol{\Theta}} \in \widehat{\Theta}_\epsilon} \exp\left(-2n_{j,a} \sum_j \sum_a (\tilde{\theta}_{j,a} - \overline{\theta}_{j,a})^2\right) \\
&\leq \sum_{\tilde{\boldsymbol{\Theta}} \in \widehat{\Theta}_\epsilon} \exp(-2\epsilon) \leq \left|\widehat{\Theta}\right| e^{-2\epsilon} \\
&\leq \left(\frac{N}{2^K} + 1\right)^{J2^K} e^{-2\epsilon}. \qquad \text{(A.10)}
\end{aligned}$$

The above result holds for any fixed $\mathbf{A}$, we apply a union bound over all the $(2^K)^N$ possible assignments of $\mathbf{A}$ and obtain

$$\mathbb{P}\left(\max_{\mathbf{A}}\left\{\sum_j \sum_a n_{j,a}(\widehat{\theta}_{j,a} - \overline{\theta}_{j,a})^2\right\} \geq \epsilon\right) \leq 2^{KN}\left(\frac{N}{2^K} + 1\right)^{J2^K} e^{-2\epsilon}. \qquad \text{(A.11)}$$

Take $\delta = 2^{KN}\left(\frac{N}{2^K} + 1\right)^{J2^K} e^{-2\epsilon}$, then $2\epsilon = N \log 2^K + J2^K \log(1 + N/2^K) - \log \delta$. This concludes the proof of Lemma 2. $\square$

Lemma 3. *Define the random variables* $X_{i,j} = R_{i,j}(1 - 2\overline{\theta}_{j,z_i})$*, and* $X = \sum_i \sum_j R_{i,j}(1 - 2\overline{\theta}_{j,z_i})$*. Note* $X_{i,j} \in [-1, 1]$*, we apply the Hoeffding's inequality to bound* $|X - \mathbb{E}[X]|$ *for any realization of* $\mathbf{A}$*:*

$$\begin{aligned}
\mathbb{P}(|X - \mathbb{E}[X]| \geq \epsilon) &\leq 2\exp\left\{-\frac{2\epsilon^2}{\sum_i \sum_j (1 - (-1))^2}\right\} \\
&\leq 2\exp\left\{-\frac{\epsilon^2}{2NJ}\right\}. \qquad \text{(A.12)}
\end{aligned}$$

With the help of Lemma 2 and Lemma 3, we next prove the following proposition.

Proposition 1. *Under the following scaling for some small positive constant* $c > 0$*,*

$$\sqrt{J} = O(N^{1-c}),$$

we have $\max_{\mathbf{A}} \left|\bar{\ell}(\mathbf{A}) - \ell(\mathbf{A})\right| = o_p(\delta_{N,J})$ *where* $\delta_{N,J} = N\sqrt{J}(\log J)^{\tilde{\epsilon}}$ *for a small positive* $\tilde{\epsilon} > 0$.

Proof. Combining the results of Lemma 2 and Lemma 3, since there are $(2^K)^N$ possible assignments of $\mathbf{A}$, we apply the union bound to obtain

$$\begin{aligned}
&\mathbb{P}(\max_{\mathbf{A}} \left|\bar{\ell}(\mathbf{A}) - \ell(\mathbf{A})\right| \geq 2\epsilon\delta_{N,J}) \\
\leq &(2^K)^N \mathbb{P}\left[\left\{\sum_j \sum_a n_{j,a}(\widehat{\theta}_{j,a} - \bar{\theta}_{j,a})^2 \geq \epsilon\delta_{N,J}\right\} \cup \{|X - \mathbb{E}[X]| \geq \epsilon\delta_{N,J}\}\right] \\
\leq &\exp\left(N\log(2^K) + J2^K \log\left(\frac{N}{2^K} + 1\right) - 2\epsilon\delta_{N,J}\right) \\
&+ 2\exp\left(N\log(2^K) - \frac{\epsilon^2\delta_{N,J}^2}{2NJ}\right).
\end{aligned} \tag{A.13}$$

For the second term on the right-hand side of the aforementioned display to converge to zero, we set $\delta_{N,J} = N\sqrt{J}(\log J)^{\tilde{\epsilon}}$ for a small positive constant $\tilde{\epsilon}$. Moreover, under this $\delta_{N,J}$, for the first term to converge to zero as N, J increase, the scaling $\sqrt{J} = O(N^{1-c})$ given in the theorem results in $\mathbb{P}(\max_{\mathbf{A}} \left|\bar{\ell}(\mathbf{A}) - \ell(\mathbf{A})\right| \geq \epsilon\delta_{N,J}) = o(1)$, which implies the result in Proposition 1. □

Step 3. (A.6) implies that $\bar{\theta}^{(\mathbf{A}^0)}_{j,z_i} = P_{i,j}$, which means that if we plug in the true latent class membership $\mathbf{A}^0$, the estimators will be the corresponding true parameters. According to this property, the following lemma shows that $\mathbf{A}^0$ minimizes the expected loss.

Lemma 4. *Note* $\bar{\ell}(\mathbf{A}^0) - \sum_i \sum_j P_{i,j}(1-P_{i,j}) = \sum_i \sum_j (P_{i,j} - \bar{\theta}_{j,z_i^0})^2 = \sum_i \sum_j (P_{i,j} - P_{i,j})^2 = 0$, *we can obtain*

$$\bar{\ell}(\mathbf{A}) - \bar{\ell}(\mathbf{A}^0) = \sum_i \sum_j (P_{i,j} - \bar{\theta}_{j,z_i})^2 \geq 0. \tag{A.14}$$

Note Lemma 4 holds for any $\mathbf{A}$, it also holds for the estimator $\widehat{\mathbf{A}}$, then

$$0 \leq \bar{\ell}(\widehat{\mathbf{A}}) - \bar{\ell}(\mathbf{A}^0) = [\bar{\ell}(\widehat{\mathbf{A}}) - \ell(\widehat{\mathbf{A}})] + [\ell(\widehat{\mathbf{A}}) - \ell(\mathbf{A}^0)] + [\ell(\mathbf{A}^0) - \bar{\ell}(\mathbf{A}^0)]. \tag{A.15}$$

Since $\widehat{\mathbf{A}} = \arg\min_{\mathbf{A}} \ell(\mathbf{A})$, we have $\ell(\widehat{\mathbf{A}}) - \ell(\mathbf{A}^0) \leq 0$. Substituting this into (A.15), we can

derive that

$$0 \le \overline{\ell}(\widehat{\mathbf{A}}) - \overline{\ell}(\mathbf{A}^0) \le 2 \sup_{\mathbf{A}} \left| \overline{\ell}(\mathbf{A}) - \ell(\mathbf{A}) \right| = o_p(\delta_{N,J}).$$

B.4 Second Half of the Proof of Theorem 1

Step 4. Motivated by Assumption 2, we define $\mathcal{J} := \{j \in [J];\ \exists k \in [K] \text{ s.t. } \boldsymbol{q}_j^0 = \boldsymbol{e}_k\}$, which represents the set of all items j that depend on only one latent attribute. Note that $\forall j \in \mathcal{J}$, $\left|\{\boldsymbol{\alpha} \circ \boldsymbol{q}_j^0; \boldsymbol{\alpha} \in \{0,1\}^K\}\right| = 2$, as $\boldsymbol{q}_j$ only contains one required latent attribute, then $\xi(\boldsymbol{q}_j^0, \boldsymbol{\alpha}) \in \{1,2\}$ for all $j \in \mathcal{J}$. Without loss of generality, we assume that if $\boldsymbol{\alpha} \circ \boldsymbol{q}_j^0 \neq \mathbf{0}$, then let $\xi(\boldsymbol{q}_j^0, \boldsymbol{\alpha}) = 2$, otherwise, let $\xi(\boldsymbol{q}_j^0, \boldsymbol{\alpha}) = 1$. We also assume that $\theta_{j,2}^0 > \theta_{j,1}^0, \forall j \in \mathcal{J}$, which aligns with the concept that subjects possessing the required latent attribute tend to perform better. For any $j \in \mathcal{J}$, define

$$N_{a,b}^j := \sum_{i=1}^N \mathbb{1}\{z_{i,j}^0 = a\}\mathbb{1}\{\widehat{z}_{i,j} = b\}, \quad (a,b) \in \{1,2\}^2. \tag{A.16}$$

Note $P_{i,j} = \mathbb{1}\{z_{i,j}^0 = 2\}\theta_{j,2}^0 + \mathbb{1}\{z_{i,j}^0 = 1\}\theta_{j,1}^0$ and $N_{2,2}^j + N_{1,2}^j = \sum_{i=1}^N \mathbb{1}\{\widehat{z}_{i,j} = 2\}$, $N_{2,1}^j + N_{1,1}^j = \sum_{i=1}^N \mathbb{1}\{\widehat{z}_{i,j} = 1\}$. By using (A.6), there is

$$\begin{aligned}
\overline{\theta}_{j,2}^{(\widehat{\mathbf{A}})} &= \frac{\sum_{i=1}^N \mathbb{1}\{\widehat{z}_{i,j} = 2\}P_{i,j}}{\sum_{i=1}^N \mathbb{1}\{\widehat{z}_{i,j} = 2\}} \\
&= \frac{\sum_{i=1}^N \mathbb{1}\{\widehat{z}_{i,j} = 2\}(\mathbb{1}\{z_{i,j}^0 = 2\}\theta_{j,2}^0 + \mathbb{1}\{z_{i,j}^0 = 1\}\theta_{j,1}^0)}{\sum_{i=1}^N \mathbb{1}\{\widehat{z}_{i,j} = 2\}} \\
&= \frac{N_{2,2}^j\theta_{j,2}^0 + N_{1,2}^j\theta_{j,1}^0}{N_{2,2}^j + N_{1,2}^j}; \\
\overline{\theta}_{j,1}^{(\widehat{\mathbf{A}})} &= \frac{N_{2,1}^j\theta_{j,2}^0 + N_{1,1}^j\theta_{j,1}^0}{N_{2,1}^j + N_{1,1}^j}.
\end{aligned} \tag{A.17}$$

Under $\widehat{\mathbf{A}}$, we impose a natural constraint $\overline{\theta}_{j,2}^{(\widehat{\mathbf{A}})} > \overline{\theta}_{j,1}^{(\widehat{\mathbf{A}})}$, $\forall j \in \mathcal{J}$ on $\widehat{\mathbf{A}}$ for identifiability purpose. This constraint does not change the previous results since $\theta_{j,2}^0 > \theta_{j,1}^0$ allows $\ell(\widehat{\mathbf{A}}) - \ell(\mathbf{A}^0) \le 0$ in (A.15) still holds, thus $\overline{\ell}(\widehat{\mathbf{A}}) - \overline{\ell}(\mathbf{A}^0) = o_p(\delta_{N,J})$ still holds under this

constraint. Combining $\overline{\theta}_{j,2}^{(\widehat{\mathbf{A}})} > \overline{\theta}_{j,1}^{(\widehat{\mathbf{A}})}$ and $\theta_{j,2}^0 > \theta_{j,1}^0$, there is

$$\begin{aligned}\overline{\theta}_{j,2}^{(\widehat{\mathbf{A}})} > \overline{\theta}_{j,1}^{(\widehat{\mathbf{A}})} &\iff (N_{2,2}^j N_{1,1}^j - N_{1,2}^j N_{2,1}^j)\theta_{j,2}^0 > (N_{2,2}^j N_{1,1}^j - N_{1,2}^j N_{2,1}^j)\theta_{j,1}^0 \\ &\iff N_{2,2}^j N_{1,1}^j > N_{2,1}^j N_{1,2}^j. \qquad \text{(A.18)}\end{aligned}$$

From (A.16), we can obtain

$$\left|\theta_{j,1}^0 - \overline{\theta}_{j,1}^{(\widehat{\mathbf{A}})}\right| = \frac{N_{2,1}^j(\theta_{j,2}^0 - \theta_{j,1}^0)}{N_{2,1}^j + N_{1,1}^j}, \quad \left|\theta_{j,2}^0 - \overline{\theta}_{j,2}^{(\widehat{\mathbf{A}})}\right| = \frac{N_{1,2}^j(\theta_{j,2}^0 - \theta_{j,1}^0)}{N_{2,2}^j + N_{1,2}^j},$$
$$\left|\theta_{j,2}^0 - \overline{\theta}_{j,1}^{(\widehat{\mathbf{A}})}\right| = \frac{N_{1,1}^j(\theta_{j,2}^0 - \theta_{j,1}^0)}{N_{2,1}^j + N_{1,1}^j}, \quad \left|\theta_{j,1}^0 - \overline{\theta}_{j,2}^{(\widehat{\mathbf{A}})}\right| = \frac{N_{2,2}^j(\theta_{j,2}^0 - \theta_{j,1}^0)}{N_{2,2}^j + N_{1,2}^j}.$$

Therefore,

$$\begin{aligned}&\overline{\ell}(\widehat{\mathbf{A}}) - \overline{\ell}(\mathbf{A}^0) \\ &= \sum_{j=1}^J \sum_{i=1}^N (P_{i,j} - \overline{\theta}_{j,\widehat{z}_i})^2 \geq \sum_{j\in\mathcal{J}} \sum_{i=1}^N (P_{i,j} - \overline{\theta}_{j,\widehat{z}_i})^2 \\ &= \sum_{j\in\mathcal{J}} \left(N_{1,1}^j(\theta_{j,1}^0 - \overline{\theta}_{j,1}^{(\widehat{\mathbf{A}})})^2 + N_{2,1}^j(\theta_{j,2}^0 - \overline{\theta}_{j,1}^{(\widehat{\mathbf{A}})})^2 + N_{1,2}^j(\theta_{j,1}^0 - \overline{\theta}_{j,2}^{(\widehat{\mathbf{A}})})^2 + N_{2,2}^j(\theta_{j,2}^0 - \overline{\theta}_{j,2}^{(\widehat{\mathbf{A}})})^2\right) \\ &= \sum_{j\in\mathcal{J}} \left(\frac{N_{1,1}^j(N_{2,1})^2 + N_{2,1}^j(N_{1,1})^2}{(N_{2,1}^j + N_{1,1}^j)^2} + \frac{N_{1,2}^j(N_{2,2})^2 + N_{2,2}^j(N_{1,2})^2}{(N_{2,2}^j + N_{1,2}^j)^2}\right)(\theta_{j,2}^0 - \theta_{j,1}^0)^2 \\ &= \sum_{j\in\mathcal{J}} \left(\frac{N_{2,1}^j N_{1,1}^j}{N_{2,1}^j + N_{1,1}^j} + \frac{N_{2,2}^j N_{1,2}^j}{N_{2,2}^j + N_{1,2}^j}\right)(\theta_{j,2}^0 - \theta_{j,1}^0)^2 \\ &\geq \delta \sum_{j\in\mathcal{J}} \left(\frac{N_{2,1}^j N_{1,1}^j}{N_{2,1}^j + N_{1,1}^j} + \frac{N_{2,2}^j N_{1,2}^j}{N_{2,2}^j + N_{1,2}^j}\right) \\ &\geq \frac{1}{2}\delta \sum_{j\in\mathcal{J}} \left(\min\{N_{2,1}^j, N_{1,1}^j\} + \min\{N_{2,2}^j, N_{1,2}^j\}\right). \qquad \text{(A.19)}\end{aligned}$$

The second inequality holds since by Assumption 1, $(\theta_{j,2}^0 - \theta_{j,1}^0)^2 \geq \delta$. One ideal scenario is that for most $j \in \mathcal{J}$, $\min\{N_{2,1}^j, N_{1,1}^j\} + \min\{N_{2,2}^j, N_{1,2}^j\} = N_{2,1}^j + N_{1,2}^j = \sum_{i=1}^N \mathbb{1}\{z_{i,j}^0 \neq \widehat{z}_{i,j}\}$, thus the misclassification error for the local latent classes could be bounded relatively tight. The following result confirms this intuition to be accurate.

Lemma 5. *Define the following random set depending on the estimated latent attribute*

profiles $\widehat{\mathbf{A}}$ *under constraint* $\overline{\theta}_{j,2}^{(\mathbf{A})} > \overline{\theta}_{j,1}^{(\mathbf{A})}, \forall j \in \mathcal{J}$*:*

$$\mathcal{J}_0 = \{j \in \mathcal{J}; N_{2,1}^j < N_{1,1}^j, N_{1,2}^j < N_{2,2}^j\};$$
$$\mathcal{J}_1 = \{j \in \mathcal{J}; N_{2,1}^j < N_{1,1}^j, N_{1,2}^j > N_{2,2}^j\};$$
$$\mathcal{J}_2 = \{j \in \mathcal{J}; N_{2,1}^j > N_{1,1}^j, N_{1,2}^j < N_{2,2}^j\},$$

then under Assumption 1 and Assumption 2, there are $|\mathcal{J}_1| = o_p(\delta_{N,J}/N)$*,* $|\mathcal{J}_2| = o_p(\delta_{N,J}/N)$

Proof. If $j \in \mathcal{J}_1$, then $\min\{N_{2,1}^j, N_{1,1}^j\} + \min\{N_{2,2}^j, N_{1,2}^j\} = N_{2,1}^j + N_{2,2}^j = \sum_{i=1}^N \mathbb{1}\{z_{i,j}^0 = 2\}$. Under Assumption 2, there is

$$\sum_{i=1}^N \mathbb{1}\{z_{i,j}^0 = 2\} \geq N\epsilon.$$

Then

$$\begin{aligned}
&\mathbb{P}\left(|\mathcal{J}_1| \geq b\frac{\delta_{N,J}}{N\delta}\right) \\
\leq\ &\mathbb{P}\left(\sum_{j\in\mathcal{J}_1} N_{2,1}^j + N_{2,2}^j \geq b\frac{\delta_{N,J}}{N\delta} \cdot N\epsilon\right) \\
\leq\ &\mathbb{P}\left(\overline{\ell}(\widehat{\mathbf{A}}) - \overline{\ell}(\mathbf{A}^0) \geq b\epsilon\delta_{N,J}\right).
\end{aligned}$$

By noting $b\epsilon$ is a constant and $\overline{\ell}(\widehat{\mathbf{A}}) - \overline{\ell}(\mathbf{A}^0) = o_p(\delta_{N,J})$, then $|\mathcal{J}_1| = o_p(\delta_{N,J}/N)$. Similar arguments give $|\mathcal{J}_2| = o_p(\delta_{N,J}/N)$, which concludes the proof of Lemma 5. □

Note (A.18) implies that $\min\{N_{2,1}^j, N_{1,1}^j\} + \min\{N_{2,2}^j, N_{1,2}^j\} \neq N_{1,1}^j + N_{2,2}^j$, $\forall j \in \mathcal{J}$, thus $\mathcal{J} = \mathcal{J}_0 \cup \mathcal{J}_1 \cup \mathcal{J}_2$. The lemma 5 implies that when δ_J goes to 0 with a mild rate, the number of elements in $\mathcal{J}_0$ dominates the number of elements in $\mathcal{J}_1 \cup \mathcal{J}_2$, thus for most $j \in \mathcal{J}$, $\min\{N_{2,1}^j, N_{1,1}^j\} + \min\{N_{2,2}^j, N_{1,2}^j\}$ should be $N_{2,1}^j + N_{1,2}^j = \sum_{i=1}^N \mathbb{1}\{z_{i,j}^0 \neq \widehat{z}_{i,j}\}$, which represents the number of subjects with the incorrectly assigned local latent classes.

Step 5. (A.19) implies that $o_p(\delta_{N,J}) \geq \overline{\ell}(\widehat{\mathbf{A}}) - \overline{\ell}(\mathbf{A}^0) \geq \delta \sum_{j\in\mathcal{J}_0} \sum_{i=1}^N \mathbb{1}\{z_{i,j}^0 \neq \widehat{z}_{i,j}\}/2$, next we focus on obtaining a lower bound of $\sum_{j\in\mathcal{J}_0} \sum_{i=1}^N \mathbb{1}\{z_{i,j}^0 \neq \widehat{z}_{i,j}\}$ to control the classification error rate $N^{-1} \sum_{i=1}^N \mathbb{1}\{\boldsymbol{\alpha}_i^0 \neq \widehat{\boldsymbol{\alpha}}_i\}$.

Motivated by Assumption 2, for each latent attribute k, denote j_k^1 the smallest integer j such that item j has a $\boldsymbol{q}$-vector $\boldsymbol{e}_k$, and denote by j_k^2 the second smallest integer j such

that $\boldsymbol{q}_j = \boldsymbol{e}_k$, etc. For positive integer m, denote

$$\mathcal{B}^m = \{j_1^m, \dots, j_K^m\}. \tag{A.20}$$

For each $k \in \{1, \dots, K\}$, denote

$$J_{\min} = \min_{1 \le k \le K} \left|\{j \in \mathcal{J}_0; \boldsymbol{q}_j^0 = \boldsymbol{e}_k\}\right|, \quad \tilde{J}_{\min} = \min_{1 \le k \le K} \left|\{j \in \mathcal{J}; \boldsymbol{q}_j^0 = \boldsymbol{e}_k\}\right|. \tag{A.21}$$

Then we have that $\mathcal{B}^m \cap \mathcal{B}^l = \emptyset$ for any $m \neq l$, thus

$$\begin{aligned}
&\sum_{i=1}^N \sum_{j \in \mathcal{J}_0} \mathbb{1}\{\xi(\boldsymbol{q}_j^0, \boldsymbol{\alpha}_i^0) \neq \xi(\boldsymbol{q}_j^0, \widehat{\boldsymbol{\alpha}}_i)\} \\
\geq &\sum_{i=1}^N \sum_{m=1}^{J_{\min}} \sum_{j \in \mathcal{B}^m} \mathbb{1}\{\xi(\boldsymbol{q}_j^0, \boldsymbol{\alpha}_i^0) \neq \xi(\boldsymbol{q}_j^0, \widehat{\boldsymbol{\alpha}}_i)\} \\
= &J_{\min} \sum_{i=1}^N \sum_{k=1}^K \mathbb{1}\{\xi(\boldsymbol{e}_k, \boldsymbol{\alpha}_i^0) \neq \xi(\boldsymbol{e}_k, \widehat{\boldsymbol{\alpha}}_i)\} \\
\geq &J_{\min} \sum_{i=1}^N \mathbb{1}\{\boldsymbol{\alpha}_i^0 \neq \widehat{\boldsymbol{\alpha}}_i\}.
\end{aligned} \tag{A.22}$$

The last inequality holds since $\sum_{k=1}^K \mathbb{1}\{\xi(\boldsymbol{e}_k, \boldsymbol{\alpha}_i^0) \neq \xi(\boldsymbol{e}_k, \widehat{\boldsymbol{\alpha}}_i)\} \geq \mathbb{1}\{\boldsymbol{\alpha}_i^0 \neq \widehat{\boldsymbol{\alpha}}_i\}$. Note (A.22) implies $o_p(\delta_{N,J}/N) \geq J_{\min} N^{-1} \sum_{i=1}^N \mathbb{1}\{\boldsymbol{\alpha}_i^0 \neq \widehat{\boldsymbol{\alpha}}_i\}$. For simplicity, denote

$$\gamma_J = \frac{\delta_{N,J}}{NJ} = \frac{(\log J)^{\tilde{\epsilon}}}{\sqrt{J}}.$$

Note (9) in Assumption 2 implies that $|\mathcal{J}|/J \geq \tilde{J}_{\min}/J \geq \delta_J$ and $J_{\min} \geq \tilde{J}_{\min} - |\mathcal{J}_1 \cup \mathcal{J}_2|$, plug these results into $o_p(\delta_{N,J}/N) \geq J_{\min} N^{-1} \sum_{i=1}^N \mathbb{1}\{\boldsymbol{\alpha}_i^0 \neq \widehat{\boldsymbol{\alpha}}_i\}$, we can obtain

$$o_p\left(\frac{\delta_{N,J}}{N}\right) + |\mathcal{J}_1 \cup \mathcal{J}_2| \geq \frac{\tilde{J}_{\min}}{N} \sum_{i=1}^N \mathbb{1}\{\boldsymbol{\alpha}_i^0 \neq \widehat{\boldsymbol{\alpha}}_i\} \geq \frac{J\delta_J}{N} \sum_{i=1}^N \mathbb{1}\{\boldsymbol{\alpha}_i^0 \neq \widehat{\boldsymbol{\alpha}}_i\}.$$

From Lemma 5 we have $|\mathcal{J}_i| = o_p(\delta_{N,J}/N)$ for $i = 1, 2$, which implies that $|\mathcal{J}_1 \cup \mathcal{J}_2| =$

$o_p(\delta_{N,J}/N)$. Plug this into the above inequality, we can conclude that

$$o_p\left(\frac{\delta_{N,J}}{N}\right) \geq \frac{J\delta_J}{N}\sum_{i=1}^{N} \mathbb{1}\{\boldsymbol{\alpha}_i^0 \neq \widehat{\boldsymbol{\alpha}}_i\},$$

which is equivalent to $N^{-1}\sum_{i=1}^{N} \mathbb{1}\{\boldsymbol{\alpha}_i^0 \neq \widehat{\boldsymbol{\alpha}}_i\} = o_p(\gamma_J/\delta_J)$. The proof of the theorem is now complete. □

The inequality (9) in Assumption 2 builds a bridge between the misclassification error for the local latent classes and the misclassification error for the latent attribute profiles $\widehat{\mathbf{A}}$ by using the inequality $\sum_{k=1}^{K} \mathbb{1}\{\xi(\boldsymbol{e}_k, \boldsymbol{\alpha}_i^0) \neq \xi(\boldsymbol{e}_k, \widehat{\boldsymbol{\alpha}}_i)\} \geq \mathbb{1}\{\boldsymbol{\alpha}_i^0 \neq \widehat{\boldsymbol{\alpha}}_i\}$.

B.5 Proof of Theorem 2

For notational simplicty, denote $n_{j,a}^0 = \sum_{i=1}^{N} \mathbb{1}\{z_{i,j}^0 = a\}$. Thus Assumption 2 implies that $\forall \boldsymbol{\alpha} \in \{0,1\}^K$, $\sum_{i=1}^{N} \mathbb{1}\{\boldsymbol{\alpha}_i^0 = \boldsymbol{\alpha}\} \geq \epsilon N$ and

$$n_{j,a}^0 \geq \frac{2^K}{2^{K_j}} N\epsilon \geq N\epsilon. \tag{A.22}$$

Recall that

$$\widehat{\theta}_{j,a} = \frac{\sum_{i=1}^{N} \mathbb{1}\{\widehat{z}_{i,j} = a\} R_{i,j}}{\sum_{i=1}^{N} \mathbb{1}\{\widehat{z}_{i,j} = a\}}.$$

Rewrite $\theta_{j,a}^0$ as similar form

$$\theta_{j,a}^0 = \frac{\sum_{i=1}^{N} \mathbb{1}\{z_{i,j}^0 = a\}\theta_{j,a}^0}{\sum_{i=1}^{N} \mathbb{1}\{z_{i,j}^0 = a\}} = \frac{\sum_{i=1}^{N} \mathbb{1}\{z_{i,j}^0 = a\} P_{i,j}}{\sum_{i=1}^{N} \mathbb{1}\{z_{i,j}^0 = a\}}.$$

By triangle inequality we have

$$
\begin{aligned}
&\max_{j,a}\left|\widehat{\theta}_{j,a}-\theta^0_{j,a}\right| \\
=&\max_{j,a}\left|\frac{\sum_{i=1}^N \mathbb{1}\{\widehat{z}_{i,j}=a\}R_{i,j}}{\sum_{i=1}^N \mathbb{1}\{\widehat{z}_{i,j}=a\}}-\frac{\sum_{i=1}^N \mathbb{1}\{z^0_{i,j}=a\}P_{i,j}}{\sum_{i=1}^N \mathbb{1}\{z^0_{i,j}=a\}}\right| \\
\le&\max_{j,a}\left|\frac{\sum_{i=1}^N \mathbb{1}\{\widehat{z}_{i,j}=a\}R_{i,j}}{\sum_{i=1}^N \mathbb{1}\{\widehat{z}_{i,j}=a\}}-\frac{\sum_{i=1}^N \mathbb{1}\{\widehat{z}_{i,j}=a\}R_{i,j}}{\sum_{i=1}^N \mathbb{1}\{z^0_{i,j}=a\}}\right| \\
&+\max_{j,a}\left|\frac{\sum_{i=1}^N \mathbb{1}\{\widehat{z}_{i,j}=a\}R_{i,j}}{\sum_{i=1}^N \mathbb{1}\{z^0_{i,j}=a\}}-\frac{\sum_{i=1}^N \mathbb{1}\{z^0_{i,j}=a\}R_{i,j}}{\sum_{i=1}^N \mathbb{1}\{z^0_{i,j}=a\}}\right| \\
&+\max_{j,a}\left|\frac{\sum_{i=1}^N \mathbb{1}\{z^0_{i,j}=a\}R_{i,j}}{\sum_{i=1}^N \mathbb{1}\{z^0_{i,j}=a\}}-\frac{\sum_{i=1}^N \mathbb{1}\{z^0_{i,j}=a\}P_{i,j}}{\sum_{i=1}^N \mathbb{1}\{z^0_{i,j}=a\}}\right| \\
\equiv&\ \mathcal{I}_1+\mathcal{I}_2+\mathcal{I}_3.
\end{aligned}
$$

We then analyze these three terms separately. For the first term,

$$
\begin{aligned}
\mathcal{I}_1 &\le \max_{j,a}\left(\sum_i \mathbb{1}\{\widehat{z}_{i,j}=a\}R_{i,j}\right)\cdot\frac{\sum_i\left|\mathbb{1}\{\widehat{z}_{i,j}=a\}-\mathbb{1}\{z^0_{i,j}=a\}\right|}{n^0_{j,a}\sum_i \mathbb{1}\{\widehat{z}_{i,j}=a\}} \\
&\le \max_{j,a}\frac{\sum_i\left|\mathbb{1}\{\widehat{z}_{i,j}=a\}-\mathbb{1}\{z^0_{i,j}=a\}\right|}{n^0_{j,a}} \\
&\le \frac{1}{\epsilon N}\sum_i \mathbb{1}\{\boldsymbol{\alpha}^0_i\neq\widehat{\boldsymbol{\alpha}}_i\}=o_p\left(\frac{\gamma_J}{\delta_J}\right).
\end{aligned}
$$

The last inequality holds since $\forall j\in[J],\ j\in[L_j],\ \sum_i\left|\mathbb{1}\{\widehat{z}_{i,j}=a\}-\mathbb{1}\{z^0_{i,j}=a\}\right|\le\sum_i \mathbb{1}\{\boldsymbol{\alpha}^0_i\neq\widehat{\boldsymbol{\alpha}}_i\}$. For the second term we have

$$
\mathcal{I}_2=\max_{j,a}\frac{\sum_i\left|R_{i,j}(\mathbb{1}\{\widehat{z}_{i,j}=a\}-\mathbb{1}\{z^0_{i,j}=a\})\right|}{n^0_{j,a}}\le\max_{j,a}\frac{\sum_i\left|\mathbb{1}\{\widehat{z}_{i,j}=a\}-\mathbb{1}\{z^0_{i,j}=a\}\right|}{n^0_{j,a}}.
$$

Due to the same reason as $\mathcal{I}_1\xrightarrow{\mathbb{P}}0$, we can also conclude that $\mathcal{I}_2=o_p(\gamma_J/\delta_J)$, thus $\mathcal{I}_1+\mathcal{I}_2=o_p(\gamma_J/\delta_J)$. For the third term, we apply Hoeffding's inequality for bounded random variables and obtain

$$
\mathbb{P}\left(\frac{\sum_i \mathbb{1}\{z^0_{i,j}=a\}(R_{i,j}-P_{i,j})}{n^0_{j,a}}\ge t\right)\le 2\exp(-2n^0_{j,a}t^2)\le 2\exp(-2\epsilon N t^2).
$$

Note the number of (j,a) pair less than or equals to $J\cdot 2^K$ under Assumption 2, we have

for $\forall t > 0$,

$$\mathbb{P}(\mathcal{I}_3 \geq t) \leq J2^{K+1} \exp(-2\epsilon N t^2). \tag{A.23}$$

Notably, 2^{K+1} remains a constant since K is fixed. By choosing $t = 1/\sqrt{N^{1-\tilde{c}}}$ for a small $\tilde{c} > 0$, the tail probability in (A.23) converges to zero when the scaling condition $\sqrt{J} = O(N^{1-c})$ holds. This implies that $\mathcal{I}_3 = o_p(1/\sqrt{N^{1-\tilde{c}}})$. Bringing together the preceding results, we can conclude that

$$\max_{j,a} \left| \widehat{\theta}_{j,a} - \theta^0_{j,a} \right| = o_p\left(\frac{\gamma_J}{\delta_J}\right) + o_p\left(\frac{1}{\sqrt{N^{1-\tilde{c}}}}\right).$$

C Proof of Theorem 3

The proof shares a similar methodology to that outlined in Appendix 1 for Theorem 1. We will focus on discussing the main differences. The principal distinction between the original GNPC method and the modified GNPC method addressed in Theorem 1 lies in the imposition of certain parameters to be strictly 0 or 1. Specifically, using the "local latent class" notation, the constraint (7) can be reformulated as follows

$$\begin{aligned} &\{\xi(\boldsymbol{q}_j, \boldsymbol{\alpha}) = 1 \Longleftrightarrow \boldsymbol{\alpha} \cdot \boldsymbol{q}_j = 0,\ \xi(\boldsymbol{q}_j, \boldsymbol{\alpha}) = L_j \Longleftrightarrow \boldsymbol{\alpha} \cdot \boldsymbol{q}_j = K_j\}\,; \\ &\left\{\theta_{j,1} = 0,\ \theta_{j,L_j} = 1\right\}. \end{aligned} \tag{B.1}$$

Then $(\widehat{\mathbf{A}}, \widehat{\boldsymbol{\Theta}}) = \arg\min_{(\mathbf{A},\boldsymbol{\Theta})} \sum_i \sum_j (R_{i,j} - \theta_{j,z_i})^2$ are defined under the constraint (B.1) in the following context. The definitions of $\ell(\mathbf{A}, \boldsymbol{\Theta}|\mathbf{R})$ and $\overline{\ell}(\mathbf{A}, \boldsymbol{\Theta})$ are the same as in (A.2) and (A.3). Given any realization of $\mathbf{A}$, denote $\widehat{\boldsymbol{\Theta}}^{(\mathbf{A})} = \arg\min_{\boldsymbol{\Theta}} \ell(\mathbf{A}, \boldsymbol{\Theta}|\mathbf{R})$ and $\overline{\boldsymbol{\Theta}}^{(\mathbf{A})} = \arg\min_{\boldsymbol{\Theta}} \overline{\ell}(\mathbf{A}, \boldsymbol{\Theta})$ under the constraint (B.1). Clearly, (A.6) still holds for $a \in \{2, \ldots, L_j - 1\}$ and $\widehat{\theta}^{(\mathbf{A})}_{j,1} = \overline{\theta}^{(\mathbf{A})}_{j,1} = 0$, $\widehat{\theta}^{(\mathbf{A})}_{j,L_j} = \overline{\theta}^{(\mathbf{A})}_{j,L_j} = 1$, thus we still have $\mathbb{E}[\widehat{\theta}_{j,a}] = \overline{\theta}_{j,a}$ for all $j \in [J],\ a \in [L_j]$

Step 1. This step mirrors Step 1 in the proof of Theorem 1, with the exception of the decomposition form. The item $\sum_{a=1}^{L_j} n_{j,a}(\widehat{\theta}_{j,a} - \overline{\theta}_{j,a})^2$ in Lemma 1 replaced by $\sum_{a=2}^{L_j-1} n_{j,a}(\widehat{\theta}_{j,a} -$

$\overline{\theta}_{j,a})^2$.

Lemma 6. *Let $(R_{i,j}; 1 \le i \le N, 1 \le j \le J)$ denote independent Bernoulli trials with parameters $(P_{i,j}; 1 \le i \le N, 1 \le j \le J)$. Under a general latent class model, given an arbitrary $\mathbf{A}$, there is*

$$\begin{aligned}
&\inf_{\mathbf{\Theta}} \mathbb{E}[\ell(\mathbf{A}, \mathbf{\Theta}|\mathbf{R})] - \inf_{\mathbf{\Theta}} \ell(\mathbf{A}, \mathbf{\Theta}|\mathbf{R}) \\
=&\sum_{j=1}^{J}\sum_{a=2}^{L_j-1} n_{j,a}(\widehat{\theta}_{j,a} - \overline{\theta}_{j,a})^2 + \sum_{i=1}^{N}\sum_{j=1}^{J}(P_{i,j} - R_{i,j})(1 - 2\overline{\theta}_{j,z_i}) \\
=&\sum_{j=1}^{J}\sum_{a=2}^{L_j-1} n_{j,a}(\widehat{\theta}_{j,a} - \overline{\theta}_{j,a})^2 + \mathbb{E}[X] - X, \qquad \text{(B.2)}
\end{aligned}$$

where $X = \sum_{j=1}^{J}\sum_{i=1}^{N} R_{i,j}(1 - 2\overline{\theta}_{j,z_i})$ is a random variable depending on $\mathbf{A}$ and L_j denotes the number of local distinct latent classes induced by $\boldsymbol{q}_j$ for item j.

Proof. Note $\overline{\ell}(\mathbf{A}) = \sum_i \sum_j (P_{i,j} - \overline{\theta}_{j,z_i})^2 + \sum_i \sum_j P_{i,j}(1 - P_{i,j})$, then we have

$$\begin{aligned}
&\overline{\ell}(\mathbf{A}) - \ell(\mathbf{A}) \\
=&\sum_i\sum_j \Big((P_{i,j} - \overline{\theta}_{j,z_i})^2 - (R_{i,j} - \widehat{\theta}_{j,z_i})^2\Big) + \sum_i\sum_j P_{i,j}(1 - P_{i,j}) \\
=&\sum_i\sum_j \Big((R_{i,j} - \overline{\theta}_{j,z_i})^2 - (R_{i,j} - \widehat{\theta}_{j,z_i})^2\Big) \\
&+ \sum_i\sum_j \big((P_{i,j} - \overline{\theta}_{j,z_i})^2 - (R_{i,j} - \overline{\theta}_{j,z_i})^2\big) + \sum_{i=1}^{N}\sum_{j=1}^{J} P_{i,j}(1 - P_{i,j}) \\
=&\sum_i\sum_j \Big((R_{i,j} - \overline{\theta}_{j,z_i})^2 - (R_{i,j} - \widehat{\theta}_{j,z_i})^2\Big) + \sum_i\sum_j (P_{i,j} - R_{i,j})(1 - 2\overline{\theta}_{j,z_i}).
\end{aligned}$$

The last equality holds because $R_{i,j}^2 = R_{i,j}$. Given a fixed $\mathbf{A}$, (A.6) implies that $\sum_{z_{i,j}=a} R_{i,j} =$

$n_{j,a}\widehat{\theta}_{j,a}$, $\sum_{z_{i,j}=a} P_{i,j} = n_{j,a}\overline{\theta}_{j,a}$ for $a \in \{2, \ldots, L_j - 1\}$, then

$$
\begin{aligned}
&\sum_i \sum_j \left((R_{i,j} - \overline{\theta}_{j,z_i})^2 - (R_{i,j} - \widehat{\theta}_{j,z_i})^2 \right) \\
=&\sum_{j=1}^{J} \sum_{a=1}^{L_j} \sum_{z_i=a} \left((R_{i,j} - \overline{\theta}_{j,a})^2 - (R_{i,j} - \widehat{\theta}_{j,a})^2 \right) \\
=&\sum_{j=1}^{J} \sum_{a=2}^{L_j-1} \sum_{z_i=a} \left(2R_{i,j}\widehat{\theta}_{j,a} - 2R_{i,j}\overline{\theta}_{j,a} + \overline{\theta}_{j,a}^2 - \widehat{\theta}_{j,a}^2 \right) \\
=&\sum_{j=1}^{J} \sum_{a=2}^{L_j-1} \left(2n_{j,a}\widehat{\theta}_{j,a}^2 - 2n_{j,a}\widehat{\theta}_{j,a}\overline{\theta}_{j,a} + n_{j,a}\overline{\theta}_{j,a}^2 - n_{j,a}\widehat{\theta}_{j,a}^2 \right) \\
=&\sum_{j=1}^{J} \sum_{a=2}^{L_j-1} n_{j,a}(\widehat{\theta}_{j,a} - \overline{\theta}_{j,a})^2. \qquad \text{(B.3)}
\end{aligned}
$$

The second equality holds since for $a \in \{1, L_j\}$, $\widehat{\theta}_{j,a} = \overline{\theta}_{j,a}$ thus $(R_{i,j} - \overline{\theta}_{j,z_i})^2 - (R_{i,j} - \widehat{\theta}_{j,z_i})^2 = 0$ when $z_i \in \{1, L_j\}$. This concludes the proof of Lemma 6. □

Step 2. This step closely parallels Step 2 in the proof of Theorem 1. Given that $\sum_{a=2}^{L_j-1} n_{j,a}(\widehat{\theta}_{j,a} - \overline{\theta}_{j,a})^2 \leq \sum_{a=1}^{L_j} n_{j,a}(\widehat{\theta}_{j,a} - \overline{\theta}_{j,a})^2$, and note that $\overline{\theta}_{j,a}$ still falls within $[0, 1]$ under the constraint (B.1), we can directly apply Lemma 2 and Lemma 3 to control $\overline{\ell}(\mathbf{A}) - \ell(\mathbf{A})$. The same scaling condition and error rate outlined in Proposition 1 remain valid.

Step 3. The main difference between this step and Step 3 in the proof of Theorem 1 derives from a fact that $\mathbf{A}^0$ does not necessarily minimize $\overline{\ell}(\mathbf{A})$. As a result, the statement that $\overline{\ell}(\mathbf{A}) \geq \overline{\ell}(\mathbf{A}^0)$ for any $\mathbf{A}$ may not be true. However, for $a \in \{2, \ldots, L_j - 1\}$, the condition $\overline{\theta}_{j,a}^{(\mathbf{A}^0)} = P_{i,j}$ continues to be valid, thus we define

$$
\begin{aligned}
S_{N,J} :=& \overline{\ell}(\mathbf{A}^0) - \sum_i \sum_j P_{i,j}(1 - P_{i,j}) = \sum_i \sum_j (P_{i,j} - \overline{\theta}_{j,z_i^0})^2 \\
=& \sum_j \sum_{a=1} \sum_{z_i^0=a} (P_{i,j} - 0)^2 + \sum_j \sum_{a=L_j} \sum_{z_i^0=a} (1 - P_{i,j})^2. \qquad \text{(B.4)}
\end{aligned}
$$

By using the same argument in (A.15), the following inequality holds

$$
-S_{N,J} \leq \overline{\ell}(\widehat{\mathbf{A}}) - \overline{\ell}(\mathbf{A}^0) \leq 2 \sup_{\mathbf{A}} \left| \overline{\ell}(\mathbf{A}) - \ell(\mathbf{A}) \right| = o_p(\delta_{N,J}).
$$

Step 4. Inspired by Assumption 2, we define $\mathcal{J} := \{j \in [J]; \exists k \in [K] \text{ s.t. } \boldsymbol{q}_j^0 = \boldsymbol{e}_k\}$, representing the set of all items j that are only dependent on one latent attribute. With the constraint (B.1) in place, when $j \in \mathcal{J}$, it holds that $\overline{\theta}_{j,a} \in \{0,1\}, \forall a \in \{1,2\}$. Correspondingly, we define $\mathcal{N}_{a,b}^j := \{i \in [N]; z_{i,j}^0 = a, \widehat{z}_{i,j} = b\}$ and $N_{a,b}^j = |\mathcal{N}_{a,b}^j|$ in the same manner as in (A.16), yielding

$$
\begin{aligned}
\overline{\ell}(\widehat{\mathbf{A}}) - \overline{\ell}(\mathbf{A}^0) &= \sum_{j=1}^{J}\sum_{i=1}^{N}(P_{i,j} - \overline{\theta}_{j,z_i})^2 - S_{N,J} \\
&\geq \sum_{j\in\mathcal{J}}\sum_{i=1}^{N}(P_{i,j} - \overline{\theta}_{j,z_i})^2 - S_{N,J} \\
&= \sum_{j\in\mathcal{J}}\left(\sum_{i\in\mathcal{N}_{1,1}^j} P_{i,j}^2 + \sum_{i\in\mathcal{N}_{1,2}^2}(P_{i,j}-1)^2 + \sum_{i\in\mathcal{N}_{2,1}^j} P_{i,j}^2 + \sum_{i\in\mathcal{N}_{2,2}^j}(P_{i,j}-1)^2\right) - S_{N,J} \\
&\geq \sum_{j\in\mathcal{J}}\left(\sum_{i\in\mathcal{N}_{1,2}^2}(P_{i,j}-1)^2 + \sum_{i\in\mathcal{N}_{2,1}^j} P_{i,j}^2\right) - S_{N,J} \\
&> \frac{1}{4}\sum_{j\in\mathcal{J}}(N_{1,2}^j + N_{2,1}^j) - S_{N,J} \\
&= \frac{1}{4}\sum_{j\in\mathcal{J}}\sum_{i=1}^{N}\mathbb{1}\{z_{i,j}^0 \neq \widehat{z}_{i,j}\} - S_{N,J}. \qquad \text{(B.5)}
\end{aligned}
$$

The last inequality holds since by Assumption 3, $1 - P_{i,j} > 1/2$ for $i \in \mathcal{N}_{1,2}^j$ and $P_{i,j} > 1/2$ for $i \in \mathcal{N}_{2,1}^j$. By the definition of $\lambda_{N,J}^2$ in (12), we have $S_{N,J} \leq NJ\lambda_{N,J}^2$. Combining with (B.5), the following inequality holds:

$$
(4NJ)\lambda_{N,J}^2 + o_p(\delta_{N,J}) \geq \sum_{j\in\mathcal{J}}\sum_{i=1}^{N}\mathbb{1}\{z_{i,j}^0 \neq \widehat{z}_{i,j}\}. \qquad \text{(B.6)}
$$

Defining $\tilde{J}_{\min} = \min_{1\leq k\leq K}\left|\{j \in \mathcal{J}; \boldsymbol{q}_j^0 = \boldsymbol{e}_k\}\right|$, note that Assumption 2 implies $|\mathcal{J}|/J \geq \tilde{J}_{\min}/J \geq \delta_J$. By using the same definition of $\mathcal{B}^m$ in (A.20) and note $\mathcal{B}^m \cap \mathcal{B}^l \neq \emptyset$ for any

$m \neq l$, then

$$
\begin{aligned}
&\sum_{j\in\mathcal{J}}\sum_{i=1}^{N} \mathbb{1}\{z_{i,j}^0 \neq \widehat{z}_{i,j}\} \\
\geq &\sum_{i=1}^{N}\sum_{m=1}^{\tilde{J}_{\min}}\sum_{j\in\mathcal{B}^m} \mathbb{1}\{\xi(\boldsymbol{q}_j^0, \boldsymbol{\alpha}_i^0) \neq \xi(\boldsymbol{q}_j^0, \widehat{\boldsymbol{\alpha}}_i)\} \\
= &\tilde{J}_{\min}\sum_{i=1}^{N}\sum_{k=1}^{K} \mathbb{1}\{\xi(\boldsymbol{e}_k, \boldsymbol{\alpha}_i^0) \neq \xi(\boldsymbol{e}_k, \widehat{\boldsymbol{\alpha}}_i)\} \\
\geq &\tilde{J}_{\min}\sum_{i=1}^{N} \mathbb{1}\{\boldsymbol{\alpha}_i^0 \neq \widehat{\boldsymbol{\alpha}}_i\} \\
\geq &(J\delta_J)\cdot\sum_{i=1}^{N} \mathbb{1}\{\boldsymbol{\alpha}_i^0 \neq \widehat{\boldsymbol{\alpha}}_i\}. \qquad (B.7)
\end{aligned}
$$

The first inequality holds since $\cup_{m=1}^{\tilde{J}_{\min}}\mathcal{B}^m \subseteq \mathcal{J}$ and the second inequality holds due to $\sum_{k=1}^{K} \mathbb{1}\{\xi(\boldsymbol{e}_k, \boldsymbol{\alpha}_i^0) \neq \xi(\boldsymbol{e}_k, \widehat{\boldsymbol{\alpha}}_i)\} \geq \mathbb{1}\{\boldsymbol{\alpha}_i^0 \neq \widehat{\boldsymbol{\alpha}}_i\}$. Combining (B.6) with (B.7) we can conclude that

$$
\frac{4\lambda_{N,J}^2}{\delta_J} + o_p\left(\frac{\gamma_J}{\delta_J}\right) \geq \frac{1}{N}\sum_{i=1}^{N} \mathbb{1}\{\boldsymbol{\alpha}_i^0 \neq \widehat{\boldsymbol{\alpha}}_i\}.
$$

This completes the proof of Theorem 3. □

There is a significant distinction between Theorem 1 and Theorem 3. According to the current literature on the consistency results for the NPC method and the GNPC method, having the true class membership minimize the expected loss is crucial for establishing clustering consistency. In the discussion of the GNPC method, the true latent attribute profiles $\mathbf{A}^0$ might not minimize the expected loss function $\bar{\ell}$ if we permit certain parameters to be zero and one. Denote $\tilde{\mathbf{A}} = \arg\min_{\mathbf{A}} \bar{\ell}(\mathbf{A})$, then $\widehat{\mathbf{A}}$ might closer to $\tilde{\mathbf{A}}$ rather than $\mathbf{A}^0$.

$\lambda_{N,J}^2$ represents the average squared distance between the true parameters and the parameters constrained to 0 or 1. From Step 3, we observe that $(NJ)\lambda_{N,J}^2 \geq \bar{\ell}(\mathbf{A}^0) - \bar{\ell}(\tilde{\mathbf{A}}) \geq 0$, implying that if $\lambda_{N,J}^2$ is small, then $\mathbf{A}^0$ nearly minimizes the expected loss $\bar{\ell}$. From this observation, $\lambda_{N,J}^2$ can be interpreted as the cost incurred by forcing some parameters to be exactly zero and one. The result given in Example 1 somewhat supports this idea.

D Proof of Example 1

Under the conditions given in Example 1, we can derive that $(P_{i,j} - \overline{\theta}_{j,z_i^0})^2 = (1/2 - \lambda)^2$. This is due to the fact that under the true latent class profiles $\mathbf{A}^0$, the pairing of $(P_{i,j}, \overline{\theta}_{j,z_i^0})$ can either be $(1/2 - \lambda, 0)$ or $(1/2 + \lambda, 1)$, thereby validating the equation (14).

For the plug-in profiles $\mathbf{A}^1$ as constructed in the example, first consider the $2M$ samples assigned to $\boldsymbol{e}_1$ in $\mathbf{A}^1$. Given that $\boldsymbol{e}_1$ contains just one latent attribute, as per the Q-matrix, when $j \in \{1, 2\}$, $\boldsymbol{e}_1$ contains half of the necessary latent attributes for items j. Therefore the true memberships for the samples assigned to $\boldsymbol{e}_1$ are half $(1, 1, 1, 1)$—with corresponding true parameter $(\lambda + 1/2)$—and half $(0, 0, 0, 0)$, with corresponding true parameter $(1/2 - \lambda)$. Thus, by (A.6), we know

$$\overline{\theta}_{j,\boldsymbol{e}_1}^{(\mathbf{A}^1)} = \frac{\sum_{i=1}^N \mathbb{1}\{z_{i,j}^{(\mathbf{A}^1)} = \boldsymbol{e}_1\} P_{i,j}}{\sum_{i=1}^N \mathbb{1}\{z_{i,j}^{(\mathbf{A}^1)} = \boldsymbol{e}_1\}} = \frac{1}{2}(1/2 + \lambda) + \frac{1}{2}(1/2 - \lambda) = \frac{1}{2},$$

when $j \in \{1, 2\}$. Next, note that $\boldsymbol{e}_1$ does not include any of the required latent attributes for items 3 and 4. Therefore $\overline{\theta}_{j,\boldsymbol{e}_1}^{(\mathbf{A}^1)} = 0$ for $j \in \{3, 4\}$.

Denote $\boldsymbol{\alpha}_i$ as the row vectors of $\mathbf{A}^1$ and $\boldsymbol{\alpha}_i^0$ as the row vectors of $\mathbf{A}^0$, then

$$
\begin{aligned}
&\sum_{\boldsymbol{\alpha}_i=\boldsymbol{e}_1}\sum_{j=1}^{4}\left(P_{i,j}-\overline{\theta}_{j,\boldsymbol{e}_1}^{(\mathbf{A}^1)}\right)^2\\
=&\sum_{\boldsymbol{\alpha}_i=\boldsymbol{e}_1}\left(\left(P_{i,1}-\frac{1}{2}\right)^2+\left(P_{i,2}-\frac{1}{2}\right)^2+P_{i,3}^2+P_{i,4}^2\right)\\
=&\sum_{\boldsymbol{\alpha}_i=\boldsymbol{e}_1,\boldsymbol{\alpha}_i^0=(1,1,1,1)}\left(\left(P_{i,1}-\frac{1}{2}\right)^2+\left(P_{i,2}-\frac{1}{2}\right)^2+P_{i,3}^2+P_{i,4}^2\right)\\
&+\sum_{\boldsymbol{\alpha}_i=\boldsymbol{e}_1,\boldsymbol{\alpha}_i^0=(0,0,0,0)}\left(\left(P_{i,1}-\frac{1}{2}\right)^2+\left(P_{i,2}-\frac{1}{2}\right)^2+P_{i,3}^2+P_{i,4}^2\right)\\
=&\sum_{\boldsymbol{\alpha}_i=\boldsymbol{e}_1,\boldsymbol{\alpha}_i^0=(1,1,1,1)}\left(2\lambda^2+2\left(\frac{1}{2}+\lambda\right)^2\right)+\sum_{\boldsymbol{\alpha}_i=\boldsymbol{e}_1,\boldsymbol{\alpha}_i^0=(0,0,0,0)}\left(2\lambda^2+2\left(\frac{1}{2}-\lambda\right)^2\right)\\
=&M\cdot\left(2\lambda^2+2\left(\frac{1}{2}+\lambda\right)^2\right)+M\cdot\left(2\lambda^2+2\left(\frac{1}{2}-\lambda\right)^2\right)\\
=&8M\cdot\left(\lambda^2+\frac{1}{8}\right)\\
=&\frac{1}{4}(NJ)\left(\lambda^2+\frac{1}{8}\right).
\end{aligned}
\tag{B.8}
$$

Here the first equality is obtained by plugging in the expressions of $\overline{\theta}_{j,\boldsymbol{e}_1}^{(\mathbf{A}^1)}$, and the last equality holds since $M = N/8$ and $J = 4$.

Since the aforementioned analysis for $\boldsymbol{e}_1$ is applicable to the other $\boldsymbol{e}_i, i \in \{2, 3, 4\}$ as well, we can insert the derived result in (B.8) into the equation (B.4). Consequently, we can deduce that the equation (15) indeed holds true. □

E Additional Simulation Results

In the main text, the simulation studies present estimation results using both the original GNPC method Chiu and Köhn (2019) and the modified GNPC method Ma et al. (2023), where the latent attributes estimated by the NPC method are used as initial values. Empirically, the NPC method provides a good initial estimate for the proficiency classes, which accelerates the convergence of both GNPC methods. Yet our theoretical results demonstrate that consistent initialization may not be necessary. In this section, we replicate the

simulation studies under all settings from the main text but randomly generate the initial values for the proficiency classes of all subjects. To mitigate the risk of local optima, we repeat the procedure five times and select the estimation with the smallest loss, $\ell(\hat{\mathbf{A}}, \hat{\mathbf{\Theta}}|\mathbf{R})$, as the final estimate. The estimation results are shown in Figure 1 and Figure 2 for data generated under the DINA model and the GDINA model, respectively.

In general, both the original GNPC method and the modified GNPC method with random initialization achieve comparable estimation errors across all scenarios to those initialized using the NPC method. These results further demonstrate that, in large-scale measurement, both the original and modified GNPC methods can consistently estimate the latent attributes without relying on NPC initialization.

We also examine the increase in computational cost for both GNPC methods when using random initialization. Specifically, we compare the average number of iterations required by both methods under random initialization versus initialization using the NPC method. The results are shown in Figure 3 and Figure 4 for data generated under the DINA model and the GDINA model, respectively. Our findings indicate a noticeable increase in computational cost with random initialization. Additionally, in more complex scenarios—such as those with higher noise levels or a greater number of attributes ($K = 5$)—the modified GNPC method requires a significantly larger number of iterations to achieve convergence. Overall, these observations highlight that the GNPC methods could consistently estimate latent attributes even with random initialization, but greater computational efficiency can be achieved when initialized with a well-chosen estimate.

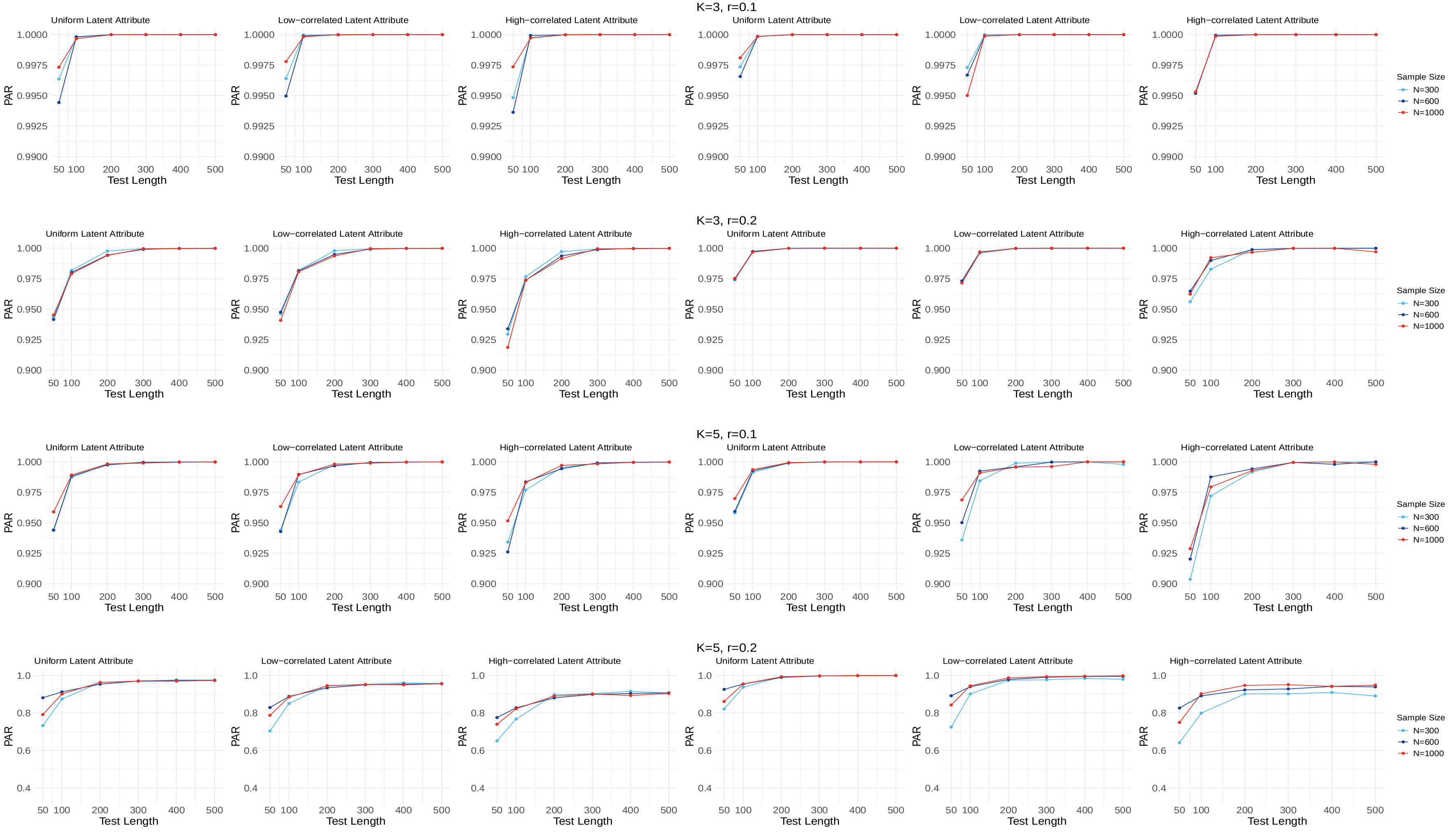

Figure 1: PARs for data generated under the DINA model with random initialization. In each row, the left three subfigures show the results of the original GNPC method, while the right three subfigures show the results of the modified GNPC method.

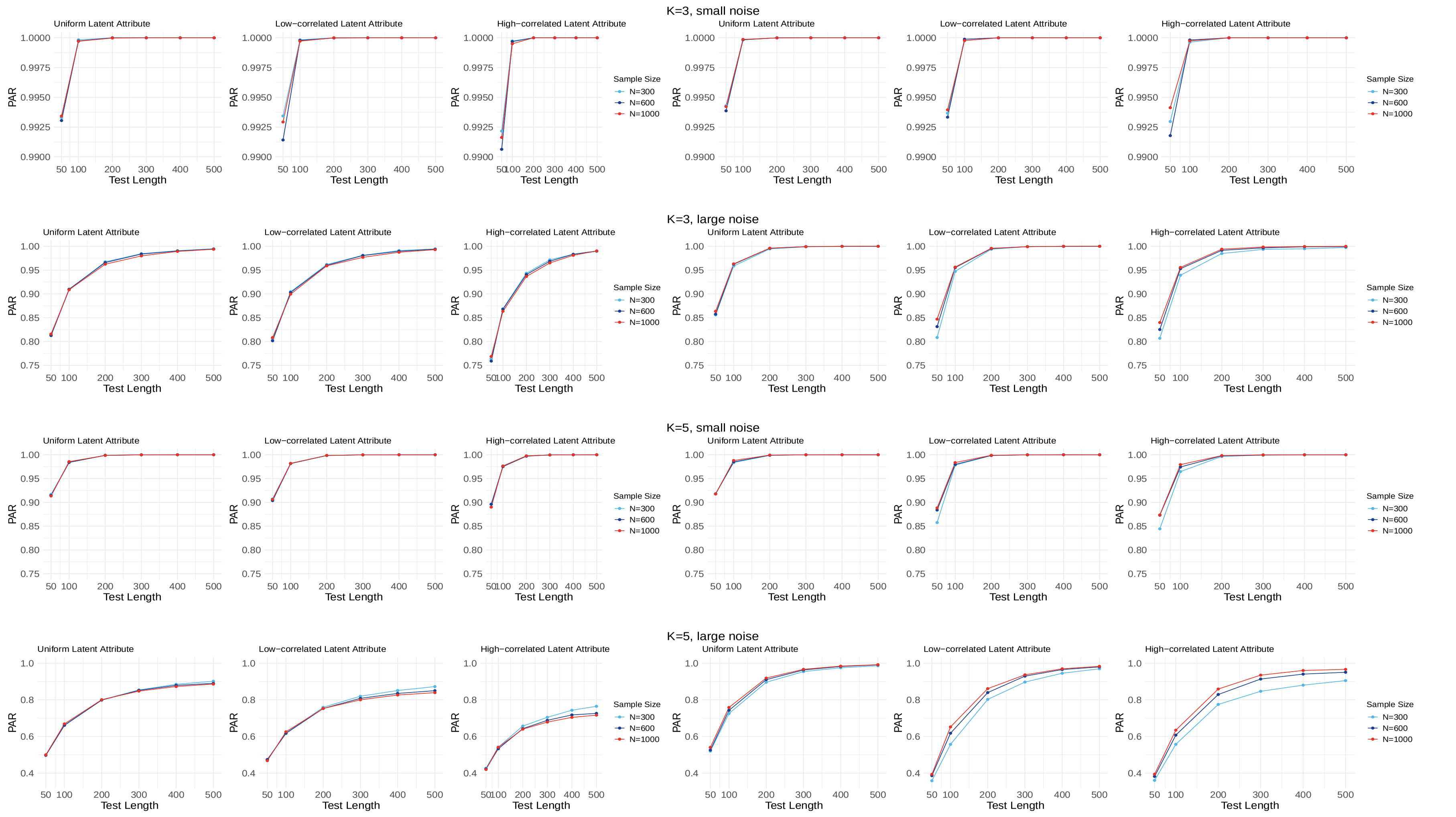

Figure 2: PARs for data generated under the GDINA model with random initialization. In each row, the left three subfigures show the results of the original GNPC method, while the right three subfigures show the results of the modified GNPC method.

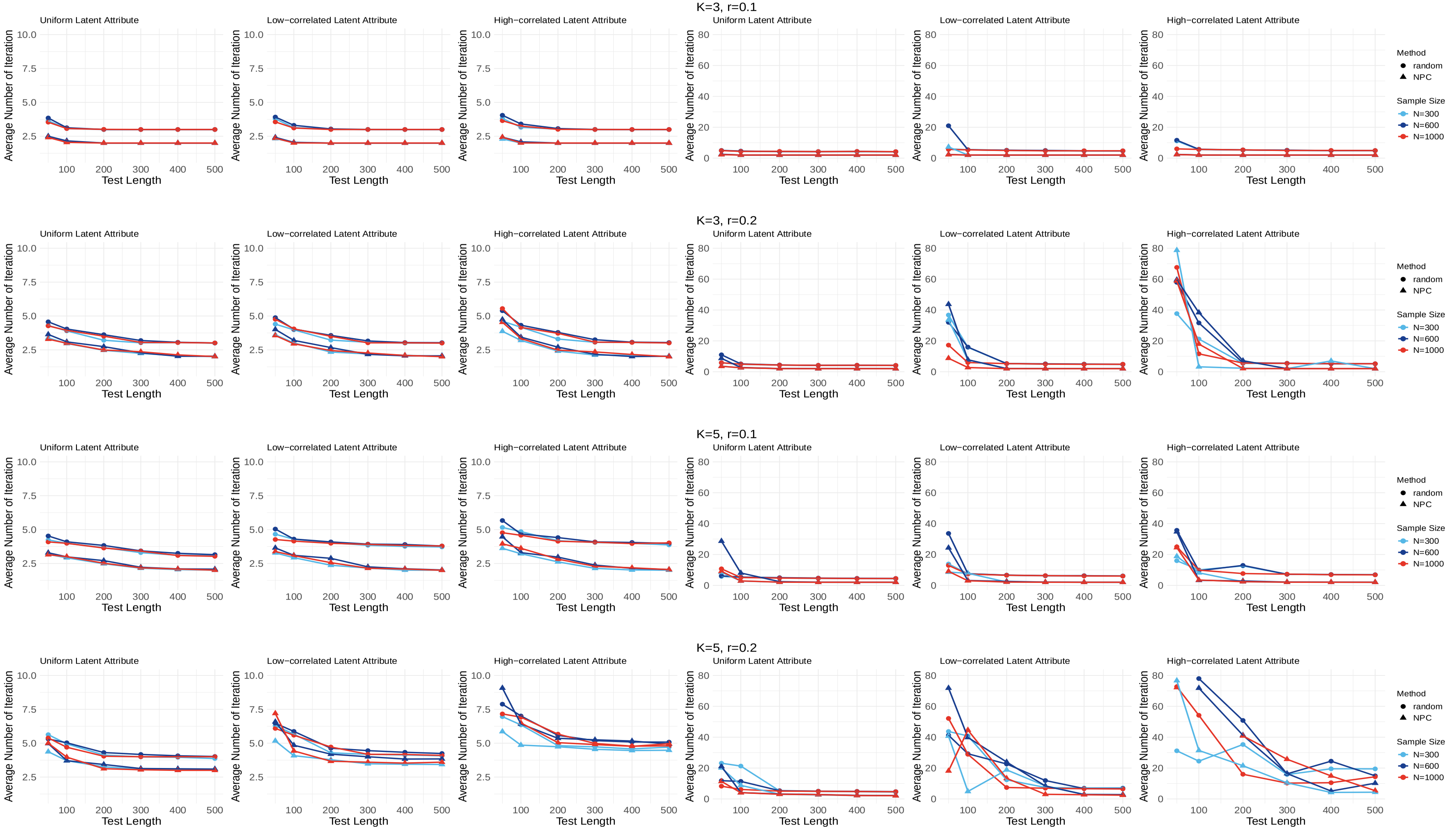

Figure 3: Average number of iteration of GNPC using random initialization and NPC initialization. Data are generated under the DINA model. In each row, the left three subfigures show the results of the original GNPC method, while the right three subfigures show the results of the modified GNPC method.

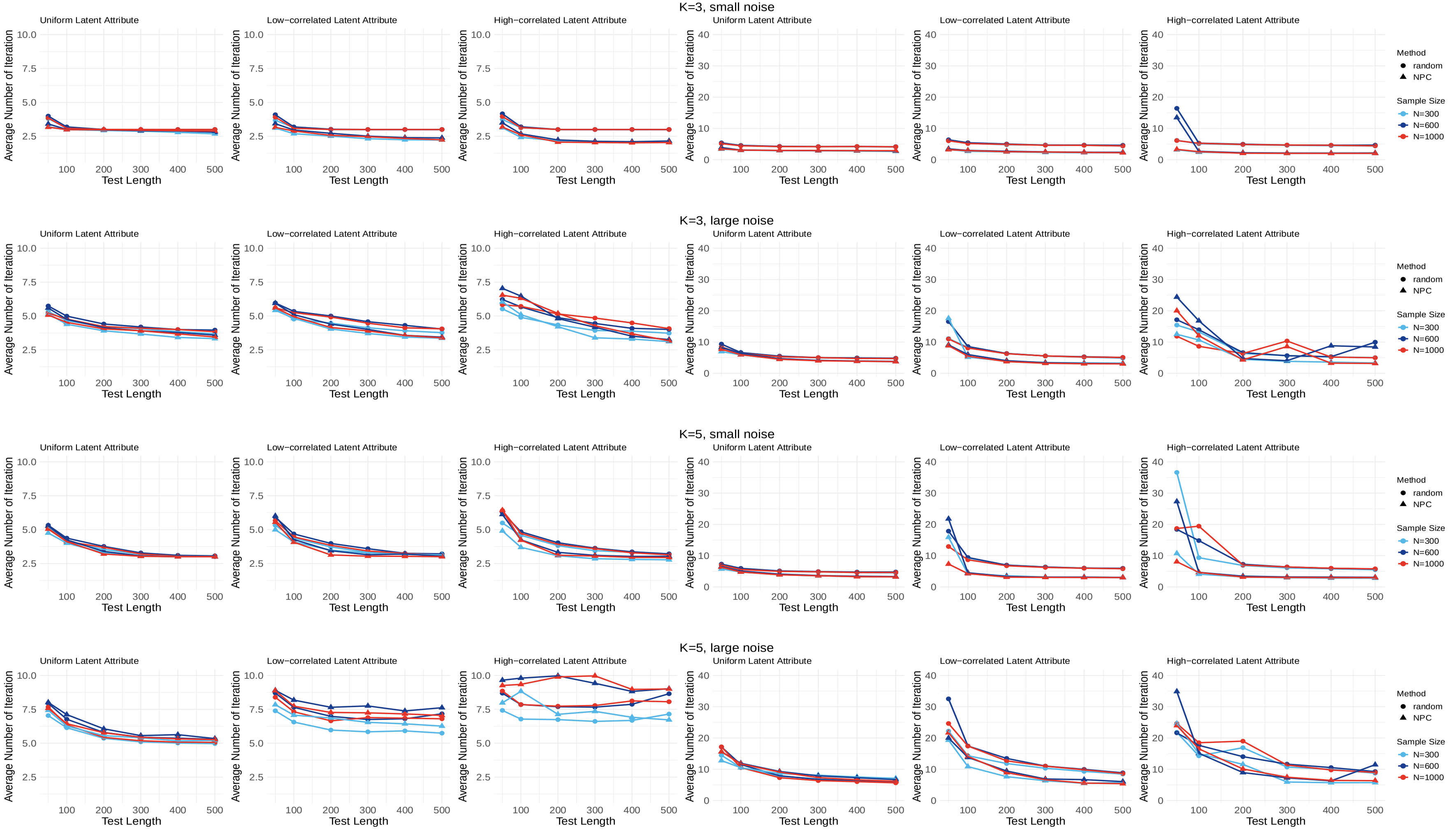

Figure 4: Average number of iteration of GNPC using random initialization and NPC initialization. Data are generated under the GDINA model. In each row, the left three subfigures show the results of the original GNPC method, while the right three subfigures show the results of the modified GNPC method.

References

Chiu, C.-Y. and Köhn, H.-F. (2019). Consistency theory for the general nonparametric classification method. *Psychometrika*, 84:830–845.

Ma, C., de la Torre, J., and Xu, G. (2023). Bridging parametric and nonparametric methods in cognitive diagnosis. *Psychometrika*, 88:51–75.
